# Capture and Protection of Environmental DNA in a Metal‐Organic Framework

**DOI:** 10.1002/smsc.202400432

**Published:** 2024-10-01

**Authors:** Laura I. FitzGerald, Erin E. Hahn, Mark Wallace, Sarah A. Stephenson, Oliver F. Berry, Cara M. Doherty

**Affiliations:** ^1^ Manufacturing CSIRO Clayton South 3169 VIC Australia; ^2^ National Research Collections Australia CSIRO Canberra 2601 ACT Australia; ^3^ National Collections and Marine Infrastructure CSIRO Black Mountain 2601 ACT Australia; ^4^ Environment CSIRO Hobart 7004 TAS Australia; ^5^ Environomics Future Science Platform National Collections and Marine Infrastructure CSIRO Crawley 6009 Western Australia Australia

**Keywords:** biodiversity, capture, environmental DNA, metal‐organic framework, quantitative polymerase chain reaction, zeolitic imidazolate framework 8

## Abstract

Environmental DNA (eDNA) is released by organisms into their surroundings, enabling non‐invasive species detection and biodiversity assessments without the need for direct observation. However, collection poses challenges due to the generally low abundance of eDNA and the presence of degradation agents, including enzymes, UV radiation, and microorganisms, rendering samples unstable. Active filtration, which is frequently used to capture eDNA in water, can be time‐consuming and cumbersome in field conditions. Herein, a filter‐free one‐pot procedure for capturing eDNA with the metal‐organic framework (MOF), zeolitic imidazolate framework 8 (ZIF‐8), is examined. The method is evaluated on 15 mL water samples from diverse sources (aquarium, river, and sea). ZIF‐8 forms in all with high capture efficiency (>98%) using spiked salmon DNA to represent eDNA. The DNA is resistant to degradation by endonucleases and UV light. In addition, it remains stable over time as a species‐specific salmon quantitative polymerase chain reaction detected genomic DNA in all samples captured with the MOF to a maximum of 28 days at 37 °C while the untreated control samples were below the assay detection limit by day 6. These results highlight the efficacy of ZIF‐8 capture in overcoming challenges associated with the preservation of eDNA obtained from aquatic environments.

## Graphical abstract

Environmental DNA (eDNA) is a rapidly growing field where species can be detected without physical observation by analyzing genetic material shed into the environment. Capture and preservation of eDNA from water is often time‐consuming and requires complex equipment. Using a simple one‐pot procedure, eDNA can be efficiently collected with a metal‐organic framework and protected from degradation.

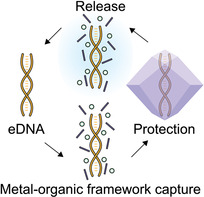

## 1 Introduction

The capture and preservation of DNA is a fundamental requirement across many scientific disciplines, including genetics, microbiology, and environmental science. Environmental DNA (eDNA) is genetic material released from organisms through cells, tissues, and waste products into the surrounding air, soil, or water.^[^ ^1^ ^]^ These remnants (both in the form of intact cells and in the form of free‐floating DNA fragments) can be collected and analyzed by methods such as quantitative polymerase chain reaction (qPCR) and high‐throughput DNA sequencing to non‐intrusively investigate biodiversity and detect species of interest.^[^ ^2^ ^]^ The field has rapidly expanded over the last two decades and often promises to deliver cheaper and more sensitive species surveillance compared to conventional methods based on physical observation.^[^ ^3^ ^]^ However, the application of eDNA methods hinges on effective collection and preservation.

Before laboratory analysis, eDNA must first be captured from an environmental substrate. This critical step, pivotal for downstream processes, can be challenging due to the generally dilute nature of eDNA. Reported total eDNA concentrations in seawater and freshwater stand at 0.2–44 and 0.5–26 ng mL^−1^, respectively.^[^ ^4^ ^]^ Within this pool of genetic material, the target sequence for species or taxonomic groups of interest may make up only a small portion. For example, most DNA collected from marine samples is microbial in origin with less than 0.0001% originating from fish.^[^ ^5^ ^]^ To detect target species within this heterogeneous mix, large volumes of water (typically from 100 mL to 10 L)^[^ ^6^, ^7^ ^]^ are filtered through membranes to increase the amount of DNA captured^[^ ^8^ ^]^ before using PCR to amplify genetic sequences of interest.^[^ ^8^ ^]^ Active water filtration, although routine, is time‐consuming, difficult to execute in turbid water, and requires specialized equipment which may limit the number of samples taken. Moreover, DNA instability necessitates careful sample preservation, often involving cold storage.^[^ ^9^ ^]^ This highlights the need for a simple and fast field‐friendly method of eDNA capture and storage uncoupled from intricate, heavy, and costly laboratory equipment.

**Figure 1:**
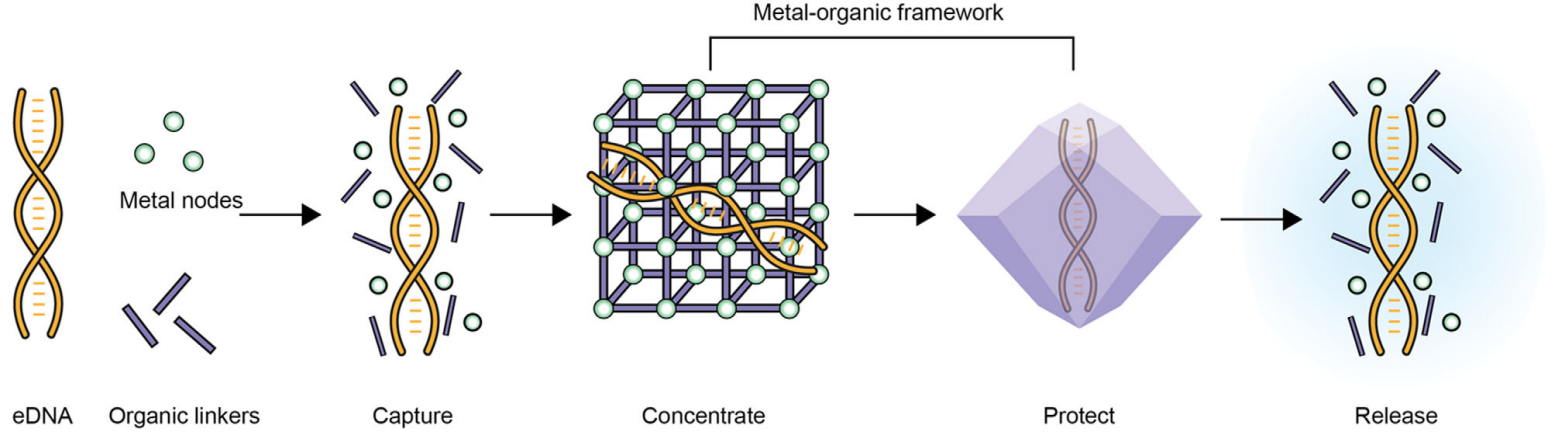
Schematic representation showing the capture of environmental DNA (eDNA) with a metal‐organic framework (MOF). Addition of salt precursors trigger formation of the MOF around the eDNA, allowing it to be concentrated and protected. The eDNA can later be released using a chelating agent.

Here, we propose the use of a metal‐organic framework (MOF) to capture, concentrate, and stabilize DNA collected in water samples (**Figure** **1**). MOFs are porous crystalline solids which coordinate organic linkers around metal nodes under specific conditions, providing an exoskeleton‐type support.^[^ ^10^ ^]^ Their use in the efficient encapsulation and protection of biomolecules against thermal and chemical challenges is well‐established.^[^ ^11^ ^]^ Specifically, MOFs have been used to encapsulate enzymes and oligonucleotides,^[^ ^12^ ^]^ plasmid DNA,^[^ ^13^, ^14^, ^15^ ^]^ chloroplasts,^[^ ^16^ ^]^ viruses,^[^ ^17^ ^]^ bacteria,^[^ ^18^ ^]^ mammalian cells,^[^ ^19^, ^20^ ^]^ and yeast,^[^ ^21^ ^]^ demonstrating their potential use on diverse biomolecules with varying sizes and surface chemistries.

The mechanism of MOF encapsulation in most of these studies occurs via a simple and rapid one‐pot process named biomimetic mineralization.^[^ ^12^ ^]^ Here, the surfaces of target molecules (such as phosphate groups on DNA) act as a nucleation site for the formation of the MOF, which then grows around the material (Figure 1).^[^ ^22^ ^]^ In this study, we used the MOF zeolitic imidazolate framework 8 (ZIF‐8), which is composed of Zn^2+^ ions and 2‐methylimdizolate linkers. ZIF‐8 reversibly encapsulates DNA^[^ ^11^, ^12^ ^]^ and its narrow aperture (≈3.4 Å) allows for physical separation from enzymes and other molecules that could potentially degrade DNA in solution.^[^ ^23^ ^]^ The key advantage of this method is its simplicity: it can be performed at room temperature, without solvents, and it only takes several minutes for the MOF to form, making it ideal for field applications. The process can also be reversed by a variety of agents including ethylenediaminetetraacetic acid (EDTA) and sodium citrate, which chelate the zinc node to trigger disassembly.^[^ ^24^ ^]^

In this study, we explore the potential of ZIF‐8 as a capturing and preserving agent within the context of aquatic DNA. Our findings reveal ZIF‐8 forms and efficiently capture DNA across multiple samples sourced from an aquarium (tank water), river, and seawater. In addition, we demonstrate the MOF can be easily disassembled, returning DNA to solution, which is then compatible with standard downstream eDNA analysis (qPCR). We also demonstrate the ability of ZIF‐8 to shield DNA from enzymatic and UV degradation over extended time periods.

## 2 Results and Discussion

### 2.1 Initial Capture of Genomic DNA in Milli‐Q Water

In a standard eDNA assay, a gene fragment typically between 50 and 400 bp^[^ ^25^ ^]^ is amplified from template DNA collected from an environmental sample. Previous encapsulation of DNA by ZIF‐8 has been performed on oligonucleotides or plasmids,^[^ ^12^, ^13^, ^14^ ^]^ which are not representative of natural sources of eDNA due to their defined sequence and homogeneous length. As an initial model for eDNA, we chose commercially available genomic *Oncorhynchus keta* (chum salmon) DNA (gDNA), which is isolated from homogenized salmon testes. Although this single‐source gDNA is also not fully representative of mixed‐source fragmented eDNA, it is available in standardized quantities and contains DNA fragments of multiple sizes, thus allowing quantitative interrogation of the protective benefits of MOFs against degradation. In addition, a qPCR assay for this organism has previously been developed which amplifies a 100 bp region from the cytochrome oxidase subunit 1 gene (COI).^[^ ^26^ ^]^ Agarose gel electrophoresis of the salmon gDNA revealed a concentrated intensity around ≈10 kb with shorter fragments <1.5 kb, similar to other reports^[^ ^27^ ^]^ (Figure S1, Supporting Information).

After the DNA source had been selected, we focused on identifying reaction conditions that would reliably generate ZIF‐8. The ZIF‐8 crystal structure can be obtained using multiple synthesis routes that incorporate different zinc salt precursors at varying ratios to the organic linker. We selected a previously described protocol that was used to encapsulate plasmid DNA by first dissolving a high concentration of 2‐methylimidazole (HmIm) in Milli‐Q (MQ) water followed by the addition of an equal volume of zinc nitrate hexahydrate (Zn(NO_3_)_2_·6H_2_O).^[^ ^14^ ^]^ A volume of 25 μL of salmon gDNA at 1 mg mL^−1^ was mixed with 125 μL of 2‐methylimidazole for 5 min before addition of 125 μL zinc nitrate hexahydrate. The final concentrations of each component were 90.9 μg mL^−1^ gDNA, 87 mg mL^−1^ 2‐methylimidazole, and 8.7 mg mL^−1^ zinc nitrate hexahydrate. After a 10 s mix with the pipette, a white precipitate began to form. Within 15 min at room temperature and without any additional agitation, the solution became fully opaque. This contrasted with when each MOF component was added in isolation and no precipitation was observed (Figure S2, Supporting Information).

**Figure 2:**
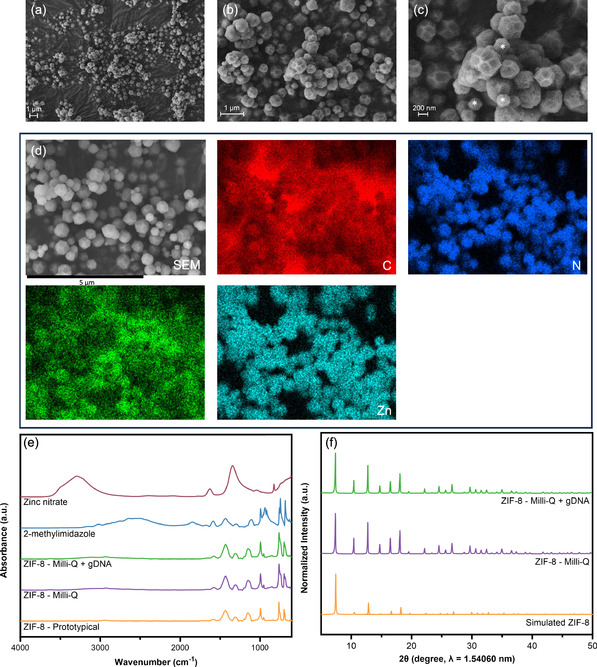
Characterization of ZIF‐8 capturing 25 μg salmon gDNA in Milli‐Q water. SEM images at a) 5000×, b) 15 000×, and c) 30 000× magnification with asterisks (*) highlighting a 200 nm subpopulation, demonstrating the mixed sizes of the sample. d) EDS elemental mapping images for carbon (C), nitrogen (N), oxygen (O), and zinc (Zn). e) FTIR spectra comparing the synthesized material to the precursors and pure ZIF‐8 without DNA. f) Simulated XRD pattern of pure ZIF‐8 compared with synthesized material with and without added gDNA.

Next, we conducted various characterization techniques to confirm the identity of the product as ZIF‐8. Characterization was performed on the precipitate that had been harvested by centrifugation, washed, and resuspended in Milli‐Q water before being vacuum dried at room temperature. Scanning electron microscopy (SEM) revealed a heterogeneous distribution of particle sizes, shapes, and topography. Predominately, particles were ≈200–500 nm with a truncated rhombic dodecahedron shape with some displaying irregular surface structures (**Figure** **2**a–c).^[^ ^28^ ^]^ A truncated cube subpopulation (≈200 nm) was also observed that possessed the same rough topography.

Further analysis by energy‐dispersive X‐ray spectroscopy (EDS) revealed a uniform distribution of the elements carbon, nitrogen, oxygen, and zinc (Figure 2d). This outcome aligns with our expectations, confirming the presence of the metal node and the constituent elements present in the linker. A Fourier‐transform infrared spectroscopy (FTIR) plot of ZIF‐8 prepared in the presence of salmon gDNA closely resembles that of ZIF‐8 synthesized without it, providing additional evidence that the white precipitate is indeed the expected MOF (Figure 2e).

Specifically, the following characteristic peaks associated with ZIF‐8 could be seen: aliphatic C–H asymmetric stretching vibration at 2928 cm^−1^, C=N stretching vibration at 1580 cm^−1^, ring stretching between 1300 and 1460 cm^−1^, and aromatic C–N stretching at 1145 cm^−1^.^[^ ^29^ ^]^ Further assignments include the presence of a peak at 694 cm^−1^ which corresponds to out‐of‐plane ring bending due to 2‐methylimidazole while C–N and C–H bending vibrations are exhibited at 994 and 759 cm^−1^ respectively.^[^ ^30^ ^]^ No major differences were seen between the ZIF‐8 spectra with or without gDNA which may reflect the low amount of gDNA relative to formed MOF. Finally, X‐ray diffraction (XRD) analysis revealed ZIF‐8 as the major phase with a degree of crystallinity of ≈71% and moderate amorphous content which can be attributed to the presence of gDNA as well as amorphous MOF material (Figure 2f). Samples prepared under identical conditions, but in the absence of gDNA, yielded highly similar results (Figure S3a–d, Supporting Information). These characterization results collectively demonstrate the robust formation of ZIF‐8 under these precursor concentrations, occurring both in the presence and in the absence of gDNA. This is particularly significant since free eDNA concentrations in environmental samples are typically low; therefore, the MOFs ability to form under such circumstances is crucial.

### 2.2 Capture Efficiency, DNA Release, and DNA Localization

**Figure 3:**
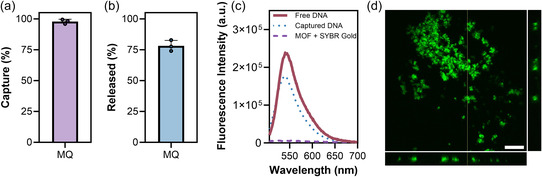
Capture of salmon gDNA in ZIF‐8 in Milli‐Q water. 275 μL samples containing final concentrations of 86 mg mL^−1^ 2‐methylimidazole, 8.7 mg mL^−1^ zinc nitrate hexahydrate, and 25 μg gDNA. Samples were incubated for 15 min before centrifuging at 10 000 g for 5 min and removing 2.2 μL of the supernatant for the SYBR Gold assay. a) Percentage capture relative to free DNA diluted to 90.9 μg mL^−1^ in Milli‐Q (MQ) and b) release following incubation of the pellet in 275 μL 100 mm EDTA for 15 min. The mean percentage (a) captured or (b) released amount of gDNA is plotted with error bars representing the standard deviation of three independent experiments (*N* = 3). c) Fluorescence spectra of free gDNA, captured gDNA, or ZIF‐8 labeled with SYBR gold. d) Orthogonal view of a confocal microscopy image showing gDNA labeled with SYBR gold in ZIF‐8 particles synthesized in Milli‐Q water. Scale bar = 30 μm.

Capture efficiency, indicative of the percentage of salmon gDNA entrapped with the MOF structure, was evaluated using SYBR Gold, a highly sensitive intercalating nucleic acid stain.^[^ ^31^ ^]^ This assessment involved comparing the fluorescence of gDNA remaining in the supernatant to the expected final concentration of gDNA if no capture occurred (90.9 μg mL^−1^). Using this approach, successful coprecipitation of 25 μg salmon gDNA with the MOF components was estimated to be over 98% (SD = 1.940, *N* = 3) (**Figure** **3**a). The precipitation only occurred when both precursors were present as there was no reduction in signal when gDNA was incubated with either zinc nitrate or 2‐ methylimidazole alone (Figure S4, Supporting Information). gDNA diluted in water showed ≈30% lower signal (Figure S4, Supporting Information) than gDNA diluted in EDTA (Figure S5, Supporting Information), likely due to the slight pH sensitivity of the dye. Therefore, we compared the supernatant to gDNA in water to determine capture efficiency and to gDNA in EDTA to calculate the percentage released.

After capture, DNA must be released for downstream processing and analysis. We chose 100 mm EDTA at pH 8 as our release agent due to its routine usage in molecular biology applications.^[^ ^32^ ^]^ High levels of this chelator may interfere with downstream qPCR.^[^ ^33^ ^]^ However we expect to remove the majority of EDTA during further processing (ethanol precipitation and extraction). Incubation of the white precipitate in 100 mm EDTA for 15 min at room temperature disassembled the MOF and the solution returned to clear. The efficiency of release, relative to the initial amount of added gDNA is estimated to be ≈78% (Figure 3b), less than the amount estimated to be captured. This difference may reflect losses of smaller particles when decanting the supernatant that was not tightly compacted in the pellet or incomplete release. Nevertheless, the majority of gDNA is retained through formation and disassembly of the MOF, and recovery may be improved with longer centrifugation times or higher speeds.

Next, we used confocal microscopy to visualize the localization of gDNA labeled with SYBR Gold. The fluorescent signal from captured and free gDNA was similar, demonstrating that SYBR gold is not quenched by the process (Figure 3c). MOFs synthesized without gDNA were also incubated with SYBR Gold, revealing no fluorescence and confirming that there was no non‐specific binding of the dye to the particles (Figure 3c). Confocal microscopy showed highly fluorescent aggregates of assorted sizes, demonstrating that the gDNA is associated with the particles either inside or adsorbed to the surface (Figure 3d and S6, Supporting Information). To further support our conclusion that DNA is captured by the MOF, we synthesized ZIF‐8 before incubating the particles with gDNA for 15 min. After centrifugation, approximately half of the gDNA remained in the supernatant (Figure S7, Supporting Information) and had not adhered to the already‐formed ZIF‐8 particles. It's important to note that this experimental setup does not directly mirror biomimetic mineralization, where a greater proportion of DNA would likely be located internally within the particles.

Collectively, these results demonstrate the efficient capture in ZIF‐8 and subsequent release using EDTA of gDNA using the SYBR Gold assay. Additionally, fluorescent microscopy provided validation of DNA association with the MOF structure.

### 2.3 Protection of Captured DNA

**Figure 4:**
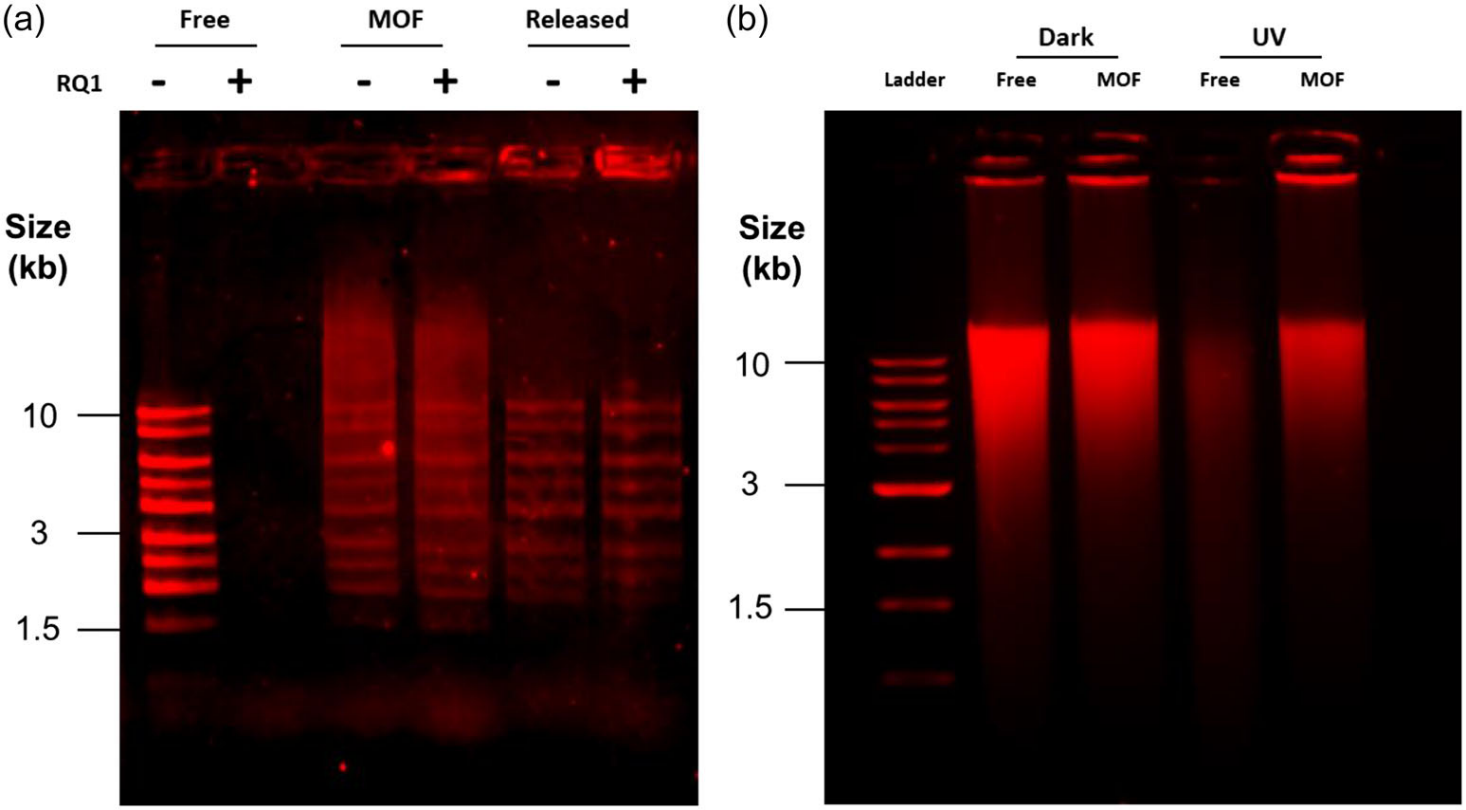
Protection provided by the MOF against endonuclease and UV degradation. a) Lanes containing 0.75 μg of 1 kb DNA ladder with (+) or without (−) DNase treatment. Lanes 1 and 2 contain free ladder while Lanes 3–6 have been captured with the ZIF‐8 MOF. Lanes 5 and 6 show the captured ladder that has been subsequently released with 100 mm EDTA. b) Lanes containing 1 μg of salmon gDNA with or without UV treatment. Lane 1 contains 0.25 μg of a 1 kb ladder. Lanes 2 and 4 contain free gDNA while 3 and 5 have been captured in the ZIF‐8 MOF. Lanes 2 and 3 were kept in the dark while 4 and 5 were subjected to 254 nm UV light for 2 h. ZIF‐8 samples were released with EDTA before loading. Both gels are 0.75% agarose containing SYBR Safe and were run at 80 V for 1 h. The ladder in (a) is from Sigma‐Aldrich while (b) is from New England Biolabs.

A variety of factors contribute to degradation of eDNA.^[^ ^34^ ^]^ Two major influences include enzymatic breakdown and UV light. We used the endonuclease, DNase I, to test the ability of ZIF‐8 to protect DNA from enzymatic digestion. Using a DNA ladder containing fragments from 0.5 to 10 kb, we compared digestion with and without ZIF‐8 capture. Unprotected ladder DNA was completely degraded following digestion with DNase (**Figure** **4**a). In contrast, captured ladder subjected to DNase digestion results in retention of the ladder's banding pattern (Figure 4a) in agreement with previous reports of the ability of ZIF‐8 to shield DNA from enzymatic breakdown.^[^ ^14^ ^]^ Similarly, we tested the ability of ZIF‐8 to protect DNA from degradation by UV light as there has been recent interest in the potential of using this MOF in UV‐shielding applications, such as in a sunscreen.^[^ ^35^, ^36^ ^]^ As we had established retention of individual bands in the previous experiment, we tested the impact of UV on the salmon gDNA which contains a broader distribution of DNA sizes. We show that heavy degradation occurs after 2 h of exposure to UV light, but that degradation is mitigated by capture with the MOF (Figure 4b).

### 2.4 Capture in Additional Water Sources

After establishing that capture, release, and protection of DNA fragments of different lengths were robust in Milli‐Q water, we next evaluated the functionality of MOFs in more environmentally relevant water sources. Water from a domestic fish tank along with samples from a local river and marine bay were obtained to further test the ability of the MOF to form under conditions with differences in pH, ionic strength, and concentrations of solids (Table S1, Supporting Information). While the pH across the three sources were similar (ranging from pH 7.3 to 7.7), the tank water had significantly less total dissolved solids and ions compared to the river and sea which may impact formation of the MOF and its morphology.^[^ ^37^ ^]^

When scaling up to 15 mL for characterization, the precursors were added directly to the mixture rather than first dissolving them in water. This approach resulted in higher final concentrations while maintaining the same precursor ratio. Characterization of the products by XRD revealed that ZIF‐8 formed in tank water in both the presence and absence of salmon gDNA (Figure S8a, Supporting Information). The FTIR spectrum also matched that of ZIF‐8 prepared by conventional methods (Figure S8b, Supporting Information). SEM imaging showed that without added gDNA, the particles that formed were rounder in shape, had a rougher surface topography, and were more uniform in size than the Milli‐Q synthesis (Figure S9a, Supporting Information). ZIF‐8 morphology is known to change when synthesis parameters are altered such as solvent, pH, and presence of other salts.^[^ ^37^, ^38^ ^]^ In the presence of gDNA however, the particles were very similar to those formed in Milli‐Q (Figure S9b, Supporting Information). Finally, EDS results confirmed the presence of zinc along with the organic linker elements (Figure S10 and S11, Supporting Information).

Synthesis of ZIF‐8 in river and seawater without addition of salmon gDNA also resulted in MOF formation which was confirmed using XRD and FTIR (Figure S12, Supporting Information). However, the particle size and surface structure were different to the patterns observed in Milli‐Q and tank water described above. The particle size that formed in the river sample was much smaller and more crystalline in appearance compared to the Milli‐Q and tank water (Figure S13a, Supporting Information) while the sea sample contained larger particles and the sample was polydisperse (Figure S13b, Supporting Information). Again, EDS analysis confirmed the expected elements for this MOF (Figure S14 and S15, Supporting Information). Taken together, these results show ZIF‐8 can be synthesized in varying water types without additional DNA added and that different water sources result in different MOF morphologies.

**Figure 5:**
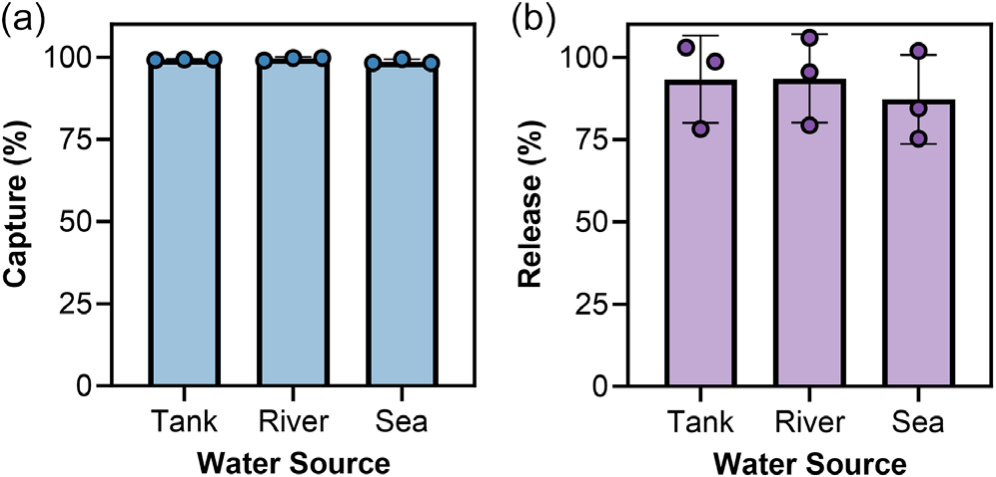
Capture and release in environmental water sources. a) Capture efficiency of 25 μg salmon gDNA spiked into 275 μL tank, river, or seawater samples containing final concentrations of 2‐methylimidazole at 86 mg mL^−1^ and zinc nitrate at 8.7 mg^−1^. SYBR Gold was added to the supernatant to determine remaining free DNA and infer precipitated quantity. b) Release of salmon gDNA with 100 mm EDTA for 15 min at room temperature compared to gDNA at the same concentration without capture. The mean in panels (a) and (b) is plotted with error bars representing the standard deviation of three independent experiments (*N* = 3).

Capture using spiked salmon gDNA was >99% efficient regardless of water source as determined by assaying for remaining DNA in the supernatant with SYBR Gold (**Figure** **5**a). Liberation of gDNA from the ZIF‐8 constructs with 100 mm EDTA was ≈91% efficient and there was no statistically significant difference (*P* = 0.8100, one‐way ANOVA) across the water types (Figure 5b).

### 2.5 Expected Recovery Using Ethanol Precipitation and a Standard Extraction Kit

The eDNA laboratory workflow we selected is based on a previously reported method that uses filters to concentrate DNA but here we concentrated via ethanol precipitation and then extracted using the commercial Qiagen DNeasy Blood & Tissue kit.^[^ ^39^ ^]^ Following water collection, a 30 mL subsample was spiked with salmon gDNA to a final concentration of ≈10 ng μL^−1^; then each sample was split into two 15 mL samples containing ≈1.5 × 10^5^ ng gDNA. One 15 mL subsample was captured with the MOF while the other was left free in solution. After incubation for the indicated time, the precipitate in the captured sample was collected by centrifugation and then released into 15 mL of 100 mm EDTA. Subsequently, both samples were subjected to identical treatment, undergoing the ethanol precipitation and extraction process.

No significant difference was observed between free DNA (≈126 ± 26 ng μL^−1^) and MOF captured DNA (≈139 ± 15 ng μL^−1^) (*P* = 0.493, unpaired *t*‐test) following extraction (Table S2, Supporting Information). This demonstrates that the DNA yield resulting from extraction with the Qiagen DNeasy Blood and Tissue kit is not impacted by the presence of MOF precursors or EDTA. The average amount of DNA after extraction was 133 ± 20 ng μL^−1^ which equates to a yield of ≈9%, highlighting inefficiencies in how we conducted the ethanol precipitation step. Efficient precipitation is achieved through higher centrifugation speeds^[^ ^40^ ^]^ than what our available equipment could reach. Thus, with minor modification, the protocol would be capable of obtaining a substantially higher yield.

### 2.6 Protection of DNA in Tank, River, and Seawater

The rate of eDNA degradation in water is highly variable and the reported time taken to fall below detectable levels by qPCR in published studies can range from hours to months depending on environmental conditions.^[^ ^41^ ^]^ Having confirmed the formation of ZIF‐8 in differing water sources and its ability to capture DNA, we aimed to replicate how this technique would be used in fieldwork. This included confirming if the MOF conferred protection from enzymatic/microbial breakdown to that DNA over time. We initially confirmed that the MOFs were stable over time in each water type as previous reports indicate that ZIF‐8 structure is unstable in aqueous conditions and biological buffers.^[^ ^42^ ^]^ We synthesized ZIF‐8 in tank, river, and seawater without adding salmon gDNA. Tank samples were incubated in the dark at 37 °C for 6 and 28 days while the river and sea samples were incubated at room temperature for 7 days. Characterization using XRD and FTIR showed ZIF‐8 was present across all water types at the experimental end point (Figure S16, Supporting Information). The sustained integrity may have been due to a difference in protocols compared to earlier studies. Our samples were intentionally left unwashed following precursor addition, potentially allowing the high concentration of 2‐methylimidazole to contribute to the increased stability of the MOF structure

To establish a baseline for our experimental system, we first investigated degradation of free salmon gDNA spiked into our water samples. Over the course of 7 days, we measured the DNA concentration by UV/visible (UV/Vis) absorbance spectrophotometry using a Nanodrop ND‐1000. We chose harsh storage conditions for the tank samples to accelerate the degradation process^[^ ^43^ ^]^ (37 °C, in an incubator). River and sea samples were stored at room temperature (c. 21 °C) on the bench to assess the capability of the MOF to preserve DNA without the need for cold storage. The concentration of DNA in Milli‐Q water remained stable for the full 7 days (Figure S17a,b, Supporting Information). In contrast, the majority of DNA was degraded after 6 days in the tank water (Figure S17a, Supporting Information), 5 days in the river water, and 7 days in the sea samples (Figure S17b, Supporting Information). This accelerated degradation in the environmental samples is most likely due to microbial activity.

**Figure 6:**
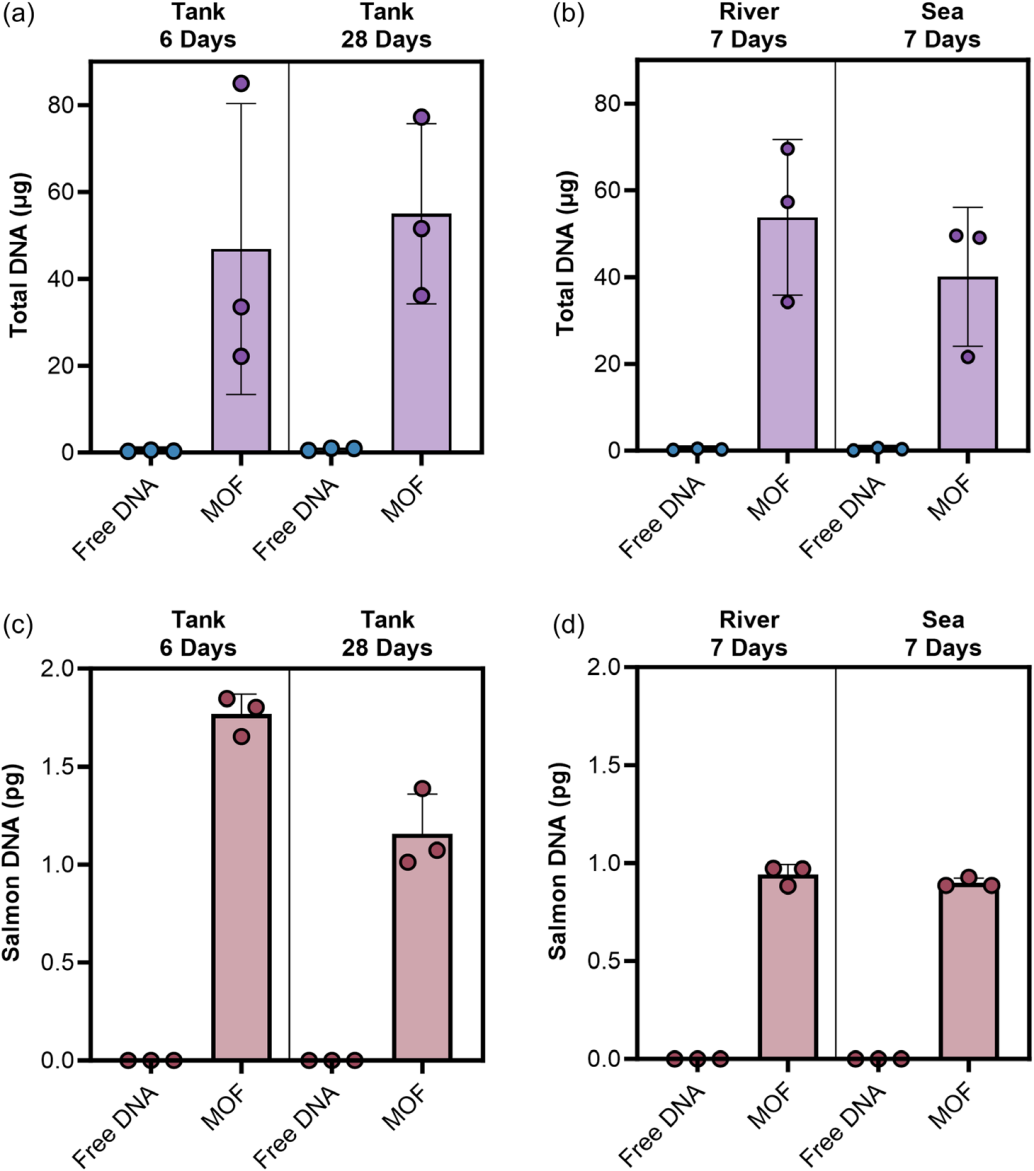
ZIF‐8 provides long‐term protection of DNA in water over time. Total DNA amount following ethanol precipitation and extraction from free or MOF‐captured water samples spiked with 10 μg mL^−1^ salmon gDNA in a) tank water incubated at 37 °C for 6 and 28 days or b) 7 days at room temperature in river or sea water measured via Nanodrop. The mean amount is plotted with error bars representing the standard deviation of three extracted samples (*N* = 3). Species‐specific salmon qPCR for the cytochrome oxidase subunit 1 gene assayed from the same c) tank or d) river and sea samples. The mean amount is plotted with error bars representing the standard deviation of three water samples analyzed in quadruplicate wells across triplicate qPCR runs (*N* = 12).

The ability of the MOF to preserve DNA over prolonged time periods in different environmental water samples was investigated. We first compared the amount of total DNA remaining in MOF samples to those that were untreated by UV/vis absorbance measurements. We again spiked water samples with salmon gDNA and incubated the tank for 6‐ and 28‐days at 37 °C and the river and sea samples for 7 days at room temperature. Measurement of the total DNA within the extracted samples revealed the remarkable efficacy of the MOF in capturing and preserving DNA over time. Tank water with DNA captured with the MOF for 6 and 28 days yielded total DNA amounts averaging over ≈50 μg while free DNA samples yielded below 1 μg per 15 mL sample (**Figure** **6**a, Table S3, and S4, Supporting Information). The variability observed across replicates is attributed to fluctuations in the amount of endogenous eDNA present alongside the spiked gDNA. Notably, the levels detected in tank samples consistently exceeded (up to 7‐fold) the yield in the Milli‐Q spiked samples, which contained only added salmon DNA and not DNA from other sources such as microorganisms (Table S2, Supporting Information). This strongly suggests that the MOF can capture and protect eDNA within the sample over time.

UV/Vis absorbance is routinely used to estimate the purity of a DNA sample. Specifically, the purity is indicated by the absorbance ratio at 260–280 nm (*A*_260_/*A*_280_ ≈ 1.8) and 260–230 nm (*A*_260_/*A*_230_ > 2.0).^[^ ^44^ ^]^ Deviations from these expected values indicate contamination from substances that also absorb UV light at these wavelengths such as proteins, detergents, and ethanol.^[^ ^45^ ^]^ Interestingly, DNA captured with the MOF within tank water exhibited higher purity following extraction compared to free DNA subjected to the exact same treatment (Table S3 and S4, Supporting Information). This contrast was not seen when DNA was extracted from Milli‐Q where the purity ratios for free and captured samples were the same (Table S2, Supporting Information). The enhanced DNA purity seen with the use of the ZIF‐8 in environmental samples might result from reduced contaminants following the pelleting of the MOF and discarding of the original supernatant before resuspension in buffer, effectively acting as a wash step.

Results using capture in river and sea samples were similar to that in tank water. Total DNA levels in untreated samples after a week at room temperature were nearly undetectable (≈6 ng μL^−1^) DNA captured with the MOF showed substantial preservation, with average concentrations of 538 ± 179 ng μL^−1^ in river water and 401 ± 160 ng μL^−1^ in sea water (Figure 6b, Table S5, and S6, Supporting Information). In addition, the *A*_260_/*A*_280_ and *A*_260_/*A*_230_ ratios again suggested MOF‐captured samples were of higher purity after extraction. This further highlights the efficacy of MOF capture in protecting DNA integrity and enhancing recovery, even in challenging environmental conditions.

Having demonstrated the effective capture and preservation of DNA in environmental samples, we assessed the suitability of the recovered DNA for downstream eDNA analysis using qPCR. We used an established qPCR assay to amplify a 100 bp region from the mitochondrial cytochrome oxidase subunit 1 gene (COI) of *Oncorhynchus keta*.^[^ ^26^ ^]^ While the absorbance results showed large amounts of DNA were present, we were uncertain if any of it was from the spiked salmon. Water samples that had been collected on three separate occasions underwent analysis on three qPCR plates, each containing quadruplicate wells, resulting in a total of 36 wells analyzed for each source and storage condition combination. Among the 288 wells, three failed to amplify, and one was identified as a significant outlier (Grubbs’ test *P* < 0.05). However, problematic wells were successfully amplified in other replicates and did not appear to be associated with any specific sample or plate.

The salmon COI gene was successfully detected in all water types in samples that had undergone MOF capture. Specifically, in the tank samples, 1.8 ± 0.10 and 1.2 ± 0.20 pg of the COI gene was detected after 6 and 28 days at 37 °C respectively (Figure 6c). In the river and sea samples, 0.94 ± 0.05 and 0.90 ± 0.02 pg were observed (Figure 6d). In contrast, the concentration of the COI gene in the free DNA samples was below the threshold of detection with the cycle threshold (*C*_t_) values falling outside the standard curve. In contrast to the variation observed between replicates in total DNA measured by absorbance (Figure 6a,b), the qPCR results exhibited significantly higher consistency. This is attributable to the target specificity of qPCR, in contrast to the variability in total eDNA in a sample. These findings underscore the effectiveness of this capture method in preserving DNA integrity over time and in challenging conditions such as elevated temperatures that environmental samples are subject to.

While the use of spiked DNA facilitated quantification and reproducibility, it does not accurately reflect typical environmental conditions.^[^ ^46^ ^]^ To address this, we analyzed water samples from the goldfish tank without adding additional DNA. We also compared our technique with eDNA collected using a cellulose acetate syringe filter,^[^ ^47^ ^]^ such as those found in commercial eDNA sampling kits.^[^ ^48^ ^]^ eDNA in 15 mL water samples was captured using either the MOF or the filter and then immediately released and extracted. The extracted samples were sent for analysis at EnviroDNA (Melbourne, Australia), for targeted species detection of goldfish.^[^ ^56^ ^]^ The test returned positive for goldfish in all water samples and across all triplicate PCR tests. While the concentration of goldfish DNA was slightly higher with the filter (ranging from 1.75 × 10^−4^ to 4.5 × 10^−4^ ng μL^−1^) compared to the MOF (ranging from 1.25 × 10^−4^ to 1.98 × 10^−4^ ng μL^−1^), both methods produced results within the same order of magnitude (**Table** **1**), demonstrating effective DNA capture under environmentally relevant concentrations. In addition, the three 28‐day room‐temperature tank samples were also analyzed for goldfish DNA. Goldfish DNA was successfully detected in all three samples subjected to MOF preservation and in all three PCR replicates for those samples, but not detected in any of the samples that had not been treated with the MOF (Table S7, Supporting Information). Although the DNA concentrations in these samples were lower than those that had been immediately released and extracted (ranging from 3.72 × 10^−5^ to 1.84 × 10^−4^ ng μL^−1^), this result demonstrates the capability of the MOF to preserve DNA over extended time periods at room temperature

**Table 1:** Results of qPCR assays for goldfish eDNA detection.

Sample	DNA Concentration [ng μL^−1^]
Filter 1	4.20 × 10^−4^
Filter 2	4.50 × 10^−4^
Filter 3	1.75 × 10^−4^
Captured 1	1.98 × 10^−4^
Captured 2	1.25 × 10^−4^
Captured 3	1.55 × 10^−4^

In this study, we have demonstrated that MOFs can capture and protect DNA in a manner that may simplify eDNA workflows. To evaluate its potential for adoption in the eDNA field, we plan to compare this new method with existing eDNA workflows in future studies. This will include comparisons with other sample preservation methods, such as Longmire's buffer, as well as a more detailed comparison with traditional filtration techniques. Finally, the toxicity of the MOF precursors may pose challenges in field applications where chemical waste disposal is difficult. This hurdle may be overcome using MOFs synthesized from more biocompatible materials like those being explored in medical applications.^[^ ^49^ ^]^ By investigating these areas further in future experiments, we aim to solidify the role of MOFs in the capture and protection of eDNA.

## 3 Conclusion

There is a growing need for improved methods to obtain and preserve genetic material, especially in remote locations and without costly or prohibitive cold storage. Applying a simple one‐pot procedure to create the MOF ZIF‐8, we demonstrate the efficient capture of DNA in solution. Through subjecting our samples to typical sources of DNA degradation such as enzymes, UV radiation, and time, we underscore the protective capabilities of ZIF‐8. Collectively, we demonstrate that the MOF captures total DNA, protects it effectively over time, and the method is the compatible with downstream qPCR. This simple process, along with its notable preservation capabilities, holds potential benefits for eDNA scientists and in various fields requiring the preservation of nucleic acids.

## 4 Experimental Section

4.1

4.1.1

##### Materials and Instrumentation

Details on all materials used and their source in addition to descriptions of instrumentation can be found in the supplementary information.

##### Preparation of Conventional ZIF‐8

Prototypical ZIF‐8 without DNA was prepared in Milli‐Q as described previously.^[^ ^50^ ^]^ Briefly, 40 mL of 160 mm 2‐methylimidazole (532.0 mg, 6.48 mmol) and 40 mL of 40 mm zinc acetate dihydrate (355.0 mg, 1.62 mmol), both in methanol, were combined. The solution was incubated for 24 h at room temperature. The precipitate was collected by centrifuging at 4000 g for 15 min and then washed twice in 40 mL methanol before being air dried without activation.

##### Standard DNA Capture in ZIF‐8

Salmon genomic DNA (gDNA) was captured with ZIF‐8 as described previously.^[^ ^14^ ^]^ Briefly, 25 μL of 1000 μg mL^−1^ salmon gDNA (25 μg) or Milli‐Q was mixed with 190 mg mL^−1^ 2‐methylimidazole (23.8 mg, 290 μmol) dissolved in 125 μL of the indicated water type (e.g., Milli‐Q, tank, river, or seawater). This was left for 5 min before addition of 19.2 mg mL^−1^ zinc nitrate hexahydrate (2.4 mg, 8.1 μmol) dissolved in 125 μL of the indicated water type. After 15 min, the white precipitate was then washed twice in 275 μL Milli‐Q H_2_O by spinning at 10 000 g for 5 min and then resuspended in the same volume with Milli‐Q H_2_O or dried under vacuum at room temperature. Larger‐scale synthesis (up to 15 mL total volume) was carried out for characterization experiments using the same ratios of DNA and precursors. Water type was varied as indicated in the text as well as the presence or absence of added salmon gDNA.

##### DNA Capture Efficiency in Milli‐Q

The capture and release efficiency of salmon gDNA in Milli‐Q was determined using the SYBR Gold assay.^[^ ^51^ ^]^ ZIF‐8 was formed using 25 μg gDNA, the supernatant was reserved, and the pellet was washed in 275 μL Milli‐Q and then spun again. The samples were then released by addition of 275 μL of 100 mm EDTA and incubated at room temperature for 15 min. Samples of free gDNA in Milli‐Q were also prepared by adding 25 μL of 1000 μg mL^−1^ salmon gDNA to 250 μL water. 2.2 μL of water, free gDNA, supernatant or released gDNA was diluted in 97.8 μL 1× tris‐EDTA buffer in a clear flat bottom 96‐well plate in triplicate. To this, 100 μL of a 2× dilution of SYBR gold in 1× tris‐EDTA buffer was added. This was incubated at room temperature for 15 min before measuring the fluorescence on the plate reader.

Capture efficiency in tank, river, and sea samples was also performed the same way. The experiment was conducted independently three times (water collected on three separate occasions).

##### Captured DNA Localization

25 μL of 1000 μg mL^−1^ salmon gDNA (25 μg) was incubated with 2.5 μL 100× SYBR Gold for 15 min and then mixed with 125 μL of 190 mg mL^−1^ 2‐methylimidazole (23.8 mg). This was left for 5 min before addition of 125 μL (19.2 mg mL^−1^) zinc nitrate hexahydrate. After 15 min, the sample was washed once with 275 μL Milli‐Q by centrifuging at 10 000 g for 5 min and resuspending in the same volume. A 2 μL drop of the suspension was then placed on a slide, covered with a coverslip, and then sealed with clear nail varnish.

Upper and lower boundaries of the Z‐stack were determined by identifying the point at which image blurring became noticeable. Approximately 20 image slices with a 1 μm step size were then acquired using a Nikon Confocal A1 system in conjunction with a Nikon Inverted Microscope (Eclipse Ti) and a Nikon Plan Apo VC 60× WI DIC N2 objective featuring a numerical aperture of 1.2. Samples were excited with a 488 nm laser, and emission signals at 525 nm with a 50 nm bandwidth were collected. A 405/488/561/640 nm dichroic mirror (Coherent Scientific) was used for laser light separation. Images were exported from NIS‐Elements (version 5.11.01, Nikon) and imported into ImageJ using the Bio‐Formats plugin (version 6.5.0).^[^ ^52^ ^]^

To test whether nonspecific association of SYBR Gold with ZIF‐8 may have quenched fluorescence, a solution with the same concentration of SYBR and precursors but without gDNA was prepared. 5 μL of this solution or 5μL of ZIF‐8‐captured gDNA was added to 495 μL Milli‐Q H_2_O in a quartz cuvette and measured using the Fluorolog‐QM.

##### ZIF‐8 Protection of DNA

The DNA protection capability of ZIF‐8 was investigated by exposing samples to a DNA‐degrading enzyme (DNase I RQ1) and UV light. A 1 kb DNA Ladder (Sigma‐Aldrich) at ≈150 ng μL^−1^ was captured with ZIF‐8 using 9 μL ladder, 45 μL 2‐methylimidazole, and 45 μL zinc nitrate hexahydrate at the concentrations and under the conditions described above. This was then split into four 20 μL samples and incubated with 1 μL DNase I (1 unit, defined as the amount required to degrade 1 μg of lambda DNA in 10 min at 37 °C in 50 μL RQ1 buffer) and 1 μL 10× RQ1 buffer or 2 μL Milli‐Q (control sample) for 10 min at 37 °C. Samples without ZIF‐8 were also prepared containing 2 μL ladder in 22 μL Milli‐Q or 2 μL ladder, 18 μL Milli‐Q, and the enzyme. RQ1 was deactivated by incubation at 75 °C for 5 min. Two samples were then released by spinning at 10 000 g for 5 min and then adding 20 μL EDTA. 4 μL 6× loading dye was added to each sample before loading on a 0.75% agarose gel, run for 1 h at 80 V, and then imaged on an Odyssey Fc Imager.

To examine UV protection, ZIF‐8 with salmon gDNA was prepared as described previously without any washes. Free gDNA without ZIF‐8 was also diluted to the same final concentration in 275 μL. The free and captured samples were transferred into wells in two separate 96‐well plates. One plate was placed in the dark while the other was centered under the lamp of a Spectroline ENF 260 C/F (6W) viewing cabinet (CM‐10) and irradiated with 254 nm UV‐C light for 2 h.^[^ ^53^ ^]^ Captured samples were then spun at 10 000 g for 5 min and resuspended in 275 μL of 100 mm EDTA for 5 min to release the DNA. 11 μL of free or released DNA was combined with 3 μL loading dye and run on a 0.75% agarose gel as described above.

##### Water Sampling

50 mL samples were collected from the surface (≈10 cm depth) into tubes from a residential fish tank (≈50 L containing 3 goldfish (*Carassius auratus*) and aquatic plants), the bank of a local river (Maribyrnong River, −37.794483, 144.910205), and from the shore of a nearby ocean bay (Hobsons Bay, −37.839825, 144.918662) in Melbourne, Australia. Salinity in Hobsons Bay was similar to seawater.^[^ ^54^ ^]^ The Maribyrnong River is variable, making it a partially mixed estuary rather than containing purely fresh or marine water.^[^ ^55^ ^]^ Sampling occurred in the morning and repeated collections were conducted with ≈7 days separation to account for variations in water composition. Water samples were stored at room temperature during transport to the laboratory (≈1 h) and then used immediately for experiments.

##### Time‐Dependent DNA Degradation

The degradation of DNA in different water sources over time was obtained by measuring the absorbance at 260 nm using a NanoDrop 1000 spectrophotometer. On the initial day (day 0), 1.4 mL of either Milli‐Q water or water collected from tank, river, or sea sources was combined with 100 μL of 1000 μg mL^−1^ salmon DNA in triplicate. The tank samples were then incubated at 37 °C to accelerate aging, while river and sea samples were kept at room temperature to closer reflect standard laboratory conditions while using the environmental samples. Subsequent absorbance measurements were taken on days 1, 5, and 7 via NanoDrop. The mean of the triplicate tubes at each time point was normalized by dividing by the mean at day 0 and then multiplied by 100 to obtain the percentage DNA remaining. The experiment was then repeated independently using water from three separate collection days.

##### Capture of Salmon and Environmental DNA in Tank, River, and Seawater

On arrival, 30 mL of the tank, river, or sea water samples were spiked with salmon gDNA to a concentration of ≈10 ng μL^−1^. These were split into two 15 mL samples in 50 mL Falcon tubes. The free DNA sample was left as is while the other was captured by dissolving 190 mg mL^−1^ 2‐methylimidazole (2.85 g, 34 mmol) directly into the 15 mL sample. After 5 min, 19.2 mg mL^−1^ zinc nitrate hexahydrate (288 mg, 0.97 mmol) was mixed. Both free and captured samples were then incubated at 37 °C for 6 or 28 days in an incubator in the dark (tank samples) or at room temperature on the bench near a window to allow access to light.

After the incubations, captured samples were released by spinning at 4000 g for 10 min, then resuspending in 100 mm EDTA, pH 8, and mixing for 1 h. DNA was precipitated from both free and released samples as described previously.^[^ ^39^ ^]^ Briefly, 1.5 mL of 3 m sodium acetate, pH 5.2, and 33 mL of absolute ethanol were added to the 15 mL water samples and stored at −20 °C overnight. The samples were centrifuged at 4000 g for 30 min before gently discarding the supernatant. Extraction of DNA was then performed using the DNeasy Blood and Tissue kit according to the manufacturer's protocol with following modifications. Samples were incubated in the Buffer ATL with added proteinase K at 56 °C for 15 min and were eluted in the last step in two lots of 50 μL Buffer AE.

To assess how much DNA was carried through the entire the precipitation and extraction process, free and captured salmon gDNA samples at day 0 in Milli‐Q were also prepared. Immediately following the 15 min incubation to form the MOF, it was released. It was then precipitated in ethanol along with the free sample and processed identically to the environmental samples.

##### Comparison to Filters

Approximately 100 mL of water from the residential fish tank containing goldfish was split into six 15 mL samples. The MOF was formed in three of these using the previously described method, while the other three were filtered using a 28 mm‐diameter, 0.45 μm pore size cellulose acetate syringe filter. For extraction, the volume of ATL buffer was increased to 540 μL and proteinase K to 60 μL to ensure complete wetting of the filter. This 600 μL mixture was passed through the filter six times using a 1 mL syringe, with the tube being returned to the heat block at 56 °C after each pass over a total of 15 min. The volumes of AL buffer and ethanol were also increased to 600 μL and the MOF samples were extracted using these increased volumes after release. The extracted samples were sent to EnviroDNA (Melbourne, Australia) for targeted species detection of goldfish DNA.^[^ ^56^ ^]^ Each sample was tested in triplicate, and two qPCR negative controls were included. Additionally, the three replicates of free and MOF samples from the tank water that had been stored at room temperature for 28 days were also analyzed.

##### qPCR Setup and Data Analysis

qPCR reaction setups were performed in a separate laboratory to DNA extractions. A 100 bp region from the cytochrome oxidase subunit 1 gene (COI) of *Oncorhynchus keta* was amplified with the following primers: 1) Forward: 5′CCGCTTTTTGTCTGAGCTGTACT; 2) Reverse: 5′ AATTTCGATCTGTGAGCAACATAGTAA

Each reaction contained 10 μL 2× PowerUp SYBR Green Master Mix for qPCR, 100 nm forward primer, 100 nm reverse primer, 2 μL template (or water for the no‐template controls (NTC)), and nuclease‐free water to make the volume up to a total of 20 μL. Thermal cycling was carried out in an Applied Biosystems 7500 Real‐Time PCR System with the following conditions: initial UDG activation at 50 °C for 2 min, dual‐lock DNA polymerase activation at 95 °C for 2 min, then 40 cycles of denaturing at 95 °C for 15 s, and annealing/extension at 60°C for 1 min. A melt curve stage was performed with a 1.6 °C s^−1^ ramp at 95 °C for 15 s, 60 °C for 1 min, and then for 0.15 °C s^−1^ at 95 °C for 15 s (Figure S18, Supporting Information).

A six‐point standard curve was constructed using tenfold serial dilutions of a 652 bp gBlock (gBlock details in Supporting Information) in quadruplicate wells (Figure S19, Supporting Information). Quadruplicate wells were also prepared for the unknown samples and NTCs in triplicate qPCR runs. The baseline was set automatically while the threshold was set manually at 0.2. Data was exported as comma‐separated value text files; then, cycle thresholds were converted to concentrations using the standard curve. Further data analysis was then performed in GraphPad Prism (version 10.2.0, GraphPad Software,San Diego, California USA).

##### Statistical Analysis

For the SYBR gold experiments, the average fluorescence intensity of water‐only controls was obtained then subtracted from the supernatant, free DNA, and released averages. The meanwas plotted with error bars representing the standard deviation. The capture efficiency and release were then calculated from

(1) $$\text{Capture}\;\left(\%\right)=\left(1-\left(\frac{{\text{FI}}_{\text{Supernatant}}}{{\text{FI}}_{\text{Free},\left(\text{MQ}\right)}}\right)\right)\times 100$$

(2) $$\text{Release}\;(\%)=\left(\frac{{\text{FI}}_{\text{Released}}}{{\text{FI}}_{\text{Free},(\text{EDTA})}}\right)\times 100\;$$

where FI_Supernatant_, FI_Free(MQ)_, FI_Free(EDTA)_ and FI_Released_ are the average fluorescence intensities of the supernatant after MOF formation, free DNA at the same concentration as added to the MOF synthesis in Milli‐Q or EDTA, the supernatant after MOF formation and centrifugation, and released sample with EDTA respectively for each independent experiment.

Statistical analyses were performed using sample sizes of *N* = 3 for fluorescence intensity measurements and *N* = 12 for qPCR experiments, where each analysis was independently repeated as specified. Differences between groups were assessed using one‐way ANOVA for multiple comparisons, with a significance level set at an alpha value of 0.05. Statistical analyses were conducted using GraphPad Prism (Version 10.2.0, GraphPad Software, San Diego, California USA).

## Conflict of Interest

The authors declare no conflict of interest.

## Author Contributions

**Laura I. FitzGerald**: Conceptualization (lead); Data curation (lead); Formal analysis (lead); Investigation (lead); Methodology (lead); Visualization (lead); Writing—original draft (lead); Writing—review & editing (lead). **Erin E. Hahn**: Conceptualization (supporting); Formal analysis (supporting); Investigation (supporting); Writing—review & editing (supporting). **Mark Wallace**: Conceptualization (supporting); Funding acquisition (supporting); Resources (supporting); Supervision (supporting); Writing—review & editing (supporting). **Sarah A. Stephenson**: Methodology (supporting); Supervision (supporting); Writing—review & editing (supporting). **Oliver F. Berry**: Conceptualization (supporting); Formal analysis (supporting); Funding acquisition (supporting); Resources (supporting); Supervision (equal); Writing—review & editing (supporting). **Cara M. Doherty**: Conceptualization (supporting); Methodology (supporting); Supervision (equal); Writing—review & editing (supporting).

## Supporting information

Supplementary Material

## Data Availability

The data that support the findings of this study are available from the corresponding author upon reasonable request.
