# Supplementary material for: Capture and Protection of Environmental DNA in a Metal‐Organic Framework
Source: Small Sci. 2024 Oct 1;4(12):2400432. doi: 10.1002/smsc.202400432 (PMC11935135; doi:10.1002/smsc.202400432)
Supplement: Supplementary file 1 — Supplementary Material [file SMSC-4-2400432-s001.pdf]

Supporting Information

Capture and Protection of Environmental DNA in a Metal-Organic Framework

Laura I. FitzGerald, Erin E. Hahn, Mark Wallace, Sarah A. Stephenson, Oliver F. Berry, Cara M. Doherty**

Materials: Milli-Q® Type 1 Ultrapure Water (MQ H_2O) was used in all experiments unless otherwise stated. Ethanol (absolute for analysis), sodium acetate trihydrate, acetic acid (glacial), zinc nitrate hexahydrate (98%), zinc acetate dihydrate (99%), 2-methylimidazole (99%), Trizma® base, deoxyribonucleic acid, sodium salt from salmon testes (D1626) and anhydrous dimethyl sulfoxide (DMSO) (≥99.9%) were obtained from Merck/Sigma-Aldrich. Ethylenediaminetetraacetic acid disodium dihydrate (EDTA) was purchased from VWR international. The salmon DNA was dissolved in MQ H_2O to a concentration of 1000 μg mL^{-1} (equivalent to a concentration of ~800 μg mL^{-1} DNA as measured via a Nanodrop 1000) and stored at -20°C. Lambda DNA Hind III Digest (D9780) and 1 kb DNA Ladder (D0428) were also purchased from Merck while 1 kb DNA ladder (N3232S) was obtained from New England Biolabs.

SYBR™ Safe DNA Gel Stain, MicroAmp™ Optical Adhesive Film, MicroAmp™ Optical 96-Well Reaction Plates, PowerUp™ SYBR™ Green Master Mix and SYBR™ Gold (10,000X Concentrate in DMSO) Nucleic Acid Gel Stain were obtained from ThermoFisher Scientific. SYBR Gold was diluted to a 100X stock in DMSO and stored at -20°C. Agarose low EEO (Agarose Standard) was purchased from AppliChem. RQ1 RNase-Free DNase and 10X RQ1 Buffer was obtained from Promega. Gel Loading Dye, Purple (6X) was purchased from New England Biolabs. The DNeasy Blood & Tissue Kit was purchased from Qiagen. PCR primers and synthetic gBlock fragments were synthesised by Integrated DNA Technologies (IDT). Stock solutions of the primers were prepared by reconstitution in nuclease free water (nfw) (IDT) to a concentration of 100 µM. 10 µM working solutions were made up as required in nfw. gBlock fragments were made up to 10 ng/µL in nuclease free water (IDT). All solutions were stored at -20°C. Minisart® NML Standard Syringe Filter, Surfactant-free Cellulose Acetate (SFCA), Pore Size 0.45 µm, 28 mm diameter were obtained from Sartorius.

gBlock Sequence: A 652-bp synthetic gene fragment (gblock) for the *Oncorhynchus keta* cytochrome oxidase subunit I (COI) gene was ordered based on the GenBank entry: EU525056.1. The sequence between the primer binding sites was modified using the IDT Codon Optimization Tool with *Salmo salar* as the selected organism. This was done so that the qPCR melt curves for the qPCR using the gBlock were slightly different from the gDNA, allowing for any contamination to be identified. Underlined sections represent primer binding sites while letters in bold show base pairs which differ from the original sequence.

cctttatttagtatttggtgcctgagccgggatagtaggcaccgccctgagcctactaattcgggcagaactaagccagccaggcgctcttctaggg
gatgaccagatctacaacgtaatcgttacagcccatgccttcgttataattttctttatagtcataccaattataatcggaggctttggaaactgattaatcc
ccctaatgatcggggcaccagatatagcattcccacgaataaataacataagcttctgactcctacctccgtccttcctcctcctttcttcatctgga
gttgaagccggcgctggtaccgggtggacagtttaccccctctagccggaaaccttgcccacgcaggagcatctgtcgacttaaccatcttctccc
tccatttagctggaatctcctcaattttgggggccattaattttattacgaccattatcaacataaaaccccagctatttctcagtaccaaacccgcttttt
gtctgagctgtactaatcac**c**gc**c**gt**tt**t**gt**t**g**ct**gc**t**g**tc**ct**t**g**cc**t**gtt**t**tggc**g**gc**c**ggtattactatgttgctcacagatcgaaatttaaacaccac
tttctttgacccagcgggcggcggagatccaattttataccaacacctc

X-ray diffraction (XRD): Samples were lightly ground in an agate mortar and pestle and loaded into low volume Si zero background sample holders. Diffractograms were obtained with a Bruker D8 Advance A25 X-ray Diffractometer operating under CuKα radiation (40kV, 40mA) equipped with a Lynx Eye XE-T detector. Samples were scanned over the 2θ range 5° to 85° with a step size of 0.02° and a count time of 1.6 second per step and were spun at 15 RPM during data collection. Analyses were performed using Bruker XRD search match program EVA™6. Crystalline phases were identified using the ICDD-JCPDS powder diffraction database. Pawley analyses were performed on the data using the Bruker TOPAS™ V6 program to determine degree of crystallinity (DOC). Background signal was described using a

combination of Chebyshev polynomial linear interpolation function and 1/x function. Cell parameters, vertical sample displacement, peak full width at half maximum and peak scale factor were all refined. DOC was calculated based on comparing the proportion of overall diffractogram intensity attributable to crystalline phases with that intensity visible as broad background humps in the data.

Attenuated total reflection Fourier transform infrared (ATR-FTIR): Spectra were collected on a Thermo Nicolet 6700 FTIR Spectrometer using 64 scans at 4 cm and analyzed using OMNIC Series Software (version 9.9.535, Thermo Scientific).

Scanning Electron Microscopy / Energy Dispersive Spectroscopy: Samples were mounted on an aluminium stub with double-sided conductive carbon tape. These samples were then iridium coated using a Cressington 208HRD sputter coater. The thickness of the iridium coating was approximately 4 nm (60 mA for 30 seconds). Conductive coating is necessary to prevent charge accumulation in an electron microscope to obtain clear images, especially for insulating material. The samples were imaged using a Zeiss Merlin FESEM (Field Emission Scanning Electron Microscope) operated in the secondary electron (SE) mode to highlight topographical features. Energy dispersive spectroscopy (EDS) was used to identify elements present within the samples. The EDS system used was AZTEC, manufactured by Oxford Instruments Pty Ltd using their X-Max Extreme 100 mm2 windowless Silicon Drift Detector (SDD) detector. This detector offers the most sensitive light element detection up to 3x increase in signal over conventional detectors at very low accelerating voltages and short working distances for optimal special EDS analysis. An accelerating voltage of 3 kV was used for imaging and 5 kV EDS analysis at a working distance of ~6.5 mm. The results are to be taken as semi-quantitative and only trends compared. The magnifications used are indicative of the scale bars shown in the images.

DNA Concentrations: DNA concentrations were obtained by UV/Visible absorbance on a NanoDrop Spectrophotometer ND-1000 using the standard nucleic acid setting.

Fluorescence Cuvette Measurements: Fluorescence spectra were collected with a Fluorolog-QM fluorometer (Horiba) with a 500 µL quartz cuvette (Hellma). The fluorescence emission between 505 – 700 nm was obtained using a 495 nm excitation and a 3 nm slit width.

Agarose gels: Agarose gels were prepared at the concentration indicated in the text using 1X tris-acetate EDTA (TAE, 40 mM Tris, 20 mM acetate and 1 mM EDTA, pH 8.6). Gels were run for 1 hour at 80 V then imaged with an Odyssey Fc Imager (LI-COR) using a 520 nm excitation and 600 nm emission with a 2-minute exposure time. Files were exported from Image Studio (LI-COR) and analyzed in ImageJ (1.52p, Wayne Rasband, National Institute of Health, USA).

Fluorescence Plate Reader: CLARIOstar Plus (BMG Labtech) was used to obtain fluorescence measurements in 96-well plates using a 495 nm 15 nm bandwidth excitation and a 537 nm 29 nm bandwidth emission wavelength.

Water Testing: pH, ion balance and total dissolved solids were measured by an independent laboratory (Envirolab, Croydon, VIC, Australia) accredited by the National Association of Testing Authorities (NATA). For each water source, a 500 mL sample was collected in a new plastic bottle and transported to the laboratory.

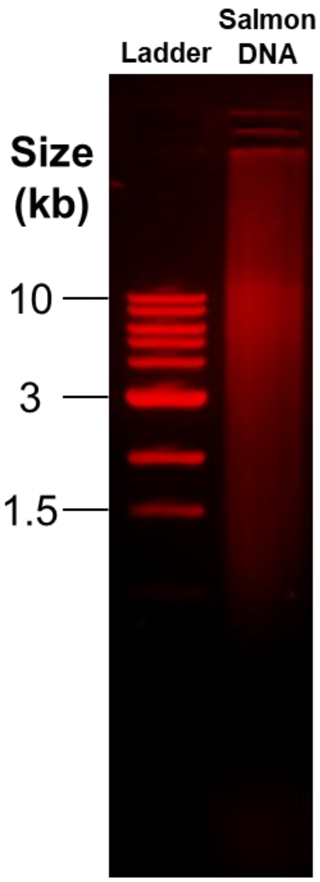

Figure S1. 1 kb ladder (New England BioLabs) and 1 µg salmon gDNA on a 1% agarose gel containing SYBR Safe run for 1 hour at 80 V.

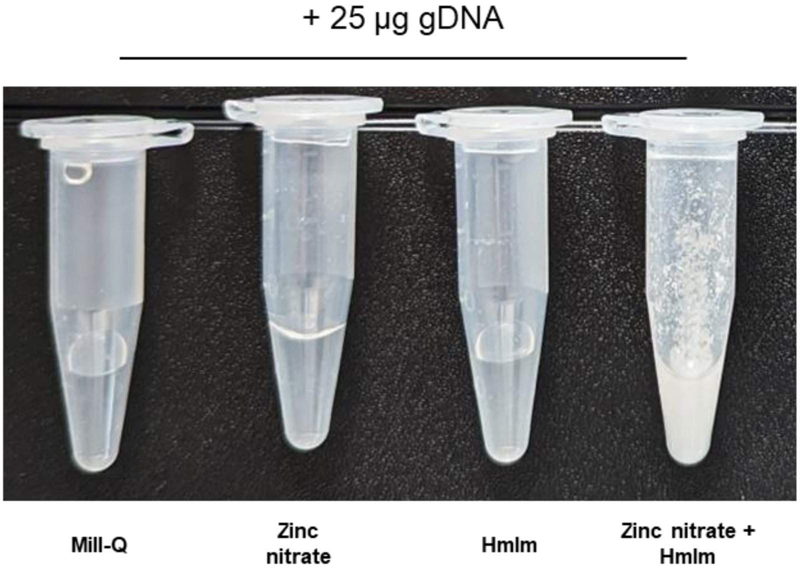

Figure S2. Precipitate formed in the presence of both ZIF-8 precursors but not when each reagent individual reagents. 25 µg gDNA in 275 µL of Milli-Q, 8.7 mg mL^{-1} zinc nitrate, 86 mg mL^{-1} 2-methylimidazole (HmIm) or both 8.7 mg mL^{-1} zinc nitrate and 86 mg mL^{-1} 2- methylimidazole.

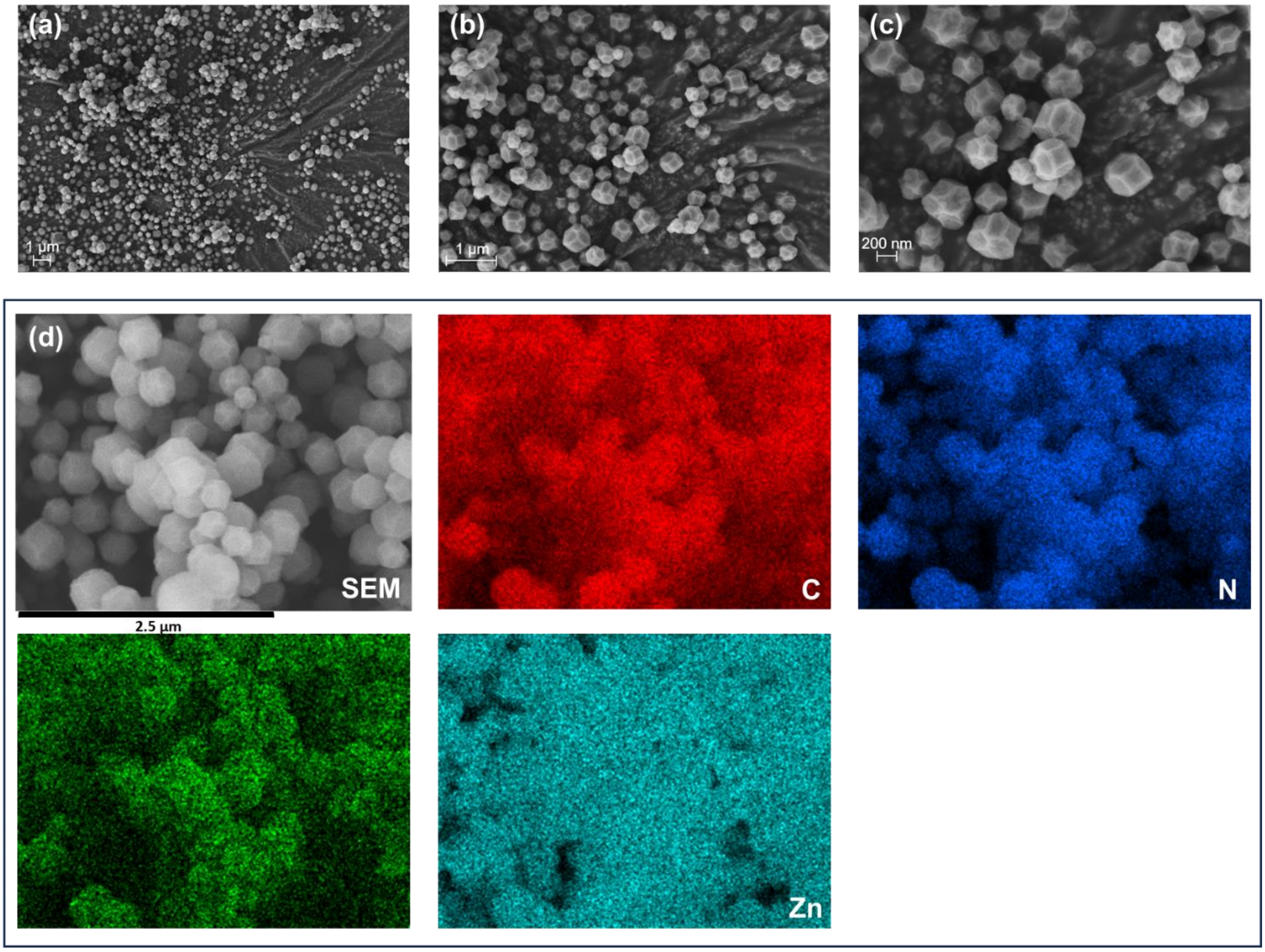

Figure S3. – Characterization of ZIF-8 synthesised in Milli-Q water without added gDNA. SEM images at (**a**) 5,000 X, (**b**) 15,000 X and (**c**) 30,000 X magnification. (**d**) EDS elemental mapping images for carbon (C), nitrogen (N), oxygen (O) and zinc (Zn).

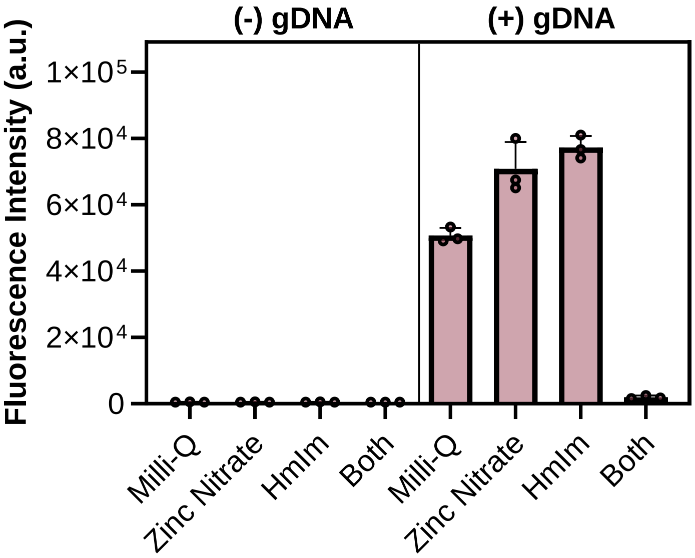

Figure S4. SYBR Gold fluorescence in the presence of ZIF-8 precursors dissolved in Milli-Q. 275 µL samples containing final concentrations of 86 mg mL^{-1} 2-methylimidazole, 8.7 mg mL^{-1} zinc nitrate hexahydrate without (-) and with (+) 25 µg gDNA. Samples were incubated for 15 minutes before spinning at 10,000 g for 5 minutes and taking 2.2 µL of the supernatant for the SYBR Gold assay. The mean fluorescence intensity is plotted with error bars representing the standard deviation of 3 independent experiments (N = 3).

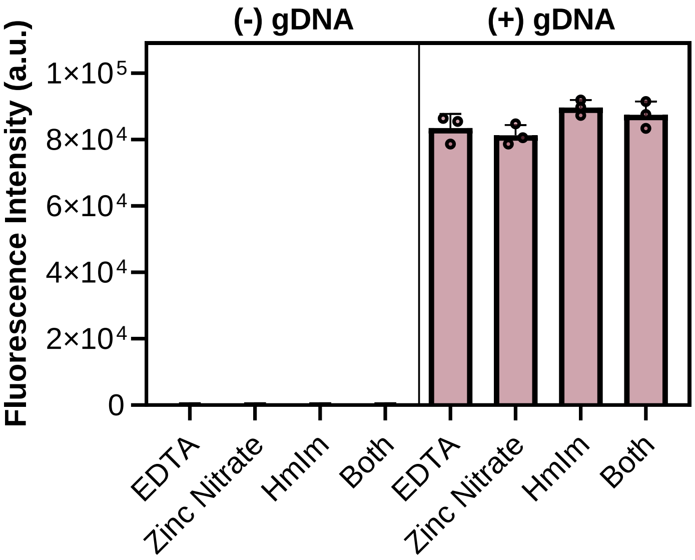

Figure S5. – SYBR Gold fluorescence in the presence of ZIF-8 precursors dissolved in 100 mM EDTA. 275 µL samples containing final concentrations of 86 mg mL^{-1} 2-methylimidazole, 8.7 mg mL^{-1} zinc nitrate hexahydrate without (-) and with (+) 25 µg gDNA. Samples were incubated for 15 minutes before spinning at 10,000 g for 5 minutes and taking 2.2 µL of the supernatant for the SYBR Gold assay. The mean fluorescence intensity is plotted with error bars representing the standard deviation of 3 independent experiments (N = 3).

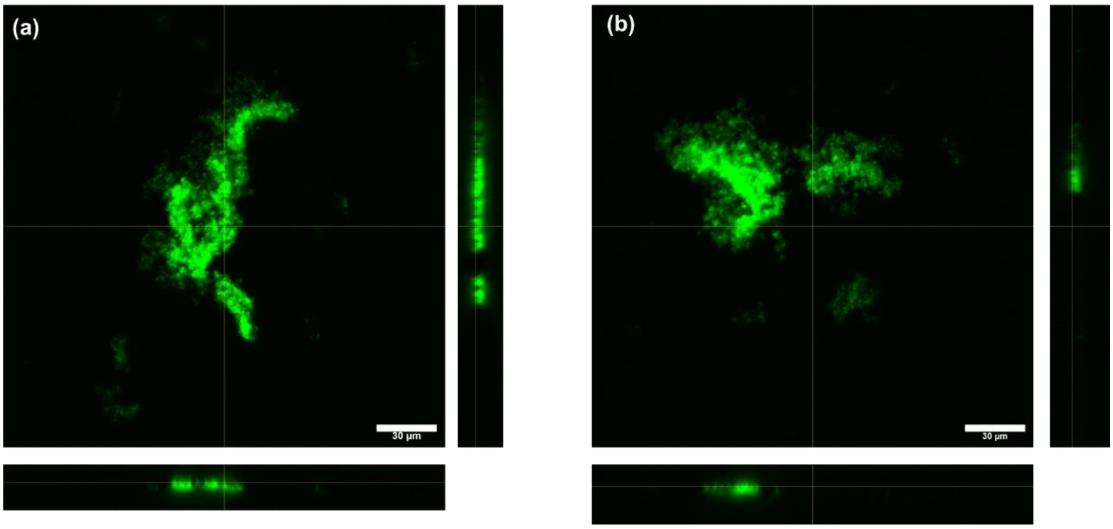

Figure S6. Additional orthogonal views from confocal microscopy images of salmon DNA captured with ZIF-8 using Milli-Q water. Scale bar = 30 µm.

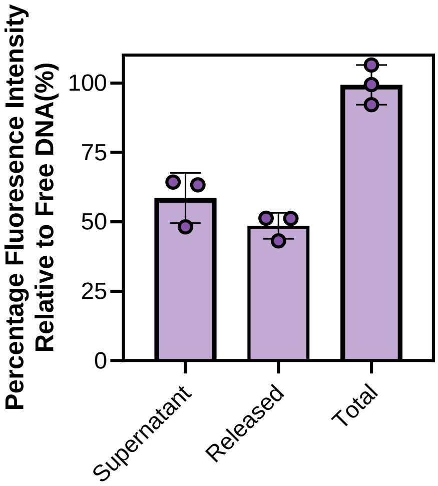

Figure S7. Adsorbance of gDNA to preformed ZIF-8. ZIF-8 formed from 25 µL Milli-Q, 125 µL of 190 mg mL^{-1} 2-methylimidazole and 125 µL 19.2 mg mL^{-1} zinc nitrate hexahydrate was washed once in 275 µL Milli-Q, resuspended in 250 µL Milli-Q then incubated with 25 µL of 1000 µg mL^{-1} salmon g DNA for 15 minutes. Samples were incubated for 15 minutes before spinning at 10,000 g for 5 minutes and taking 2.2 µL of the supernatant for the SYBR Gold assay. The remaining supernatant was discarded, and the pellet was resuspended in 275 µL of 100 mM EDTA for 15 minutes to release the genomic DNA (gDNA). Subsequently, 2.2 µL was extracted for the assay. The mean fluorescence intensity is plotted with error bars representing the standard deviation of 3 independent experiments (N = 3).

Table S1. pH, ion balance and total dissolved solids testing of tank, river, and seawater samples.

Test	Units	Tank	River	Sea
pH	pH units	7.3	7.6	7.7
Total Dissolved Solids	mg L^{-1}	240	35,000	42,000
Dissolved Calcium	mg L^{-1}	37	400	470
Dissolved Potassium	mg L^{-1}	5.9	400	360
Dissolved Sodium	mg L^{-1}	11	7,200	9,400
Dissolved Magnesium	mg L^{-1}	6.5	920	1,200
Hydroxide Alkalinity (OH^{-1}) as $CaCO_3$	mg L^{-1}	< 5	< 5	< 5
Bicarbonate Alkalinity as $CaCO_3$	mg L^{-1}	31	110	120
Carbonate Alkalinity as $CaCO_3$	mg L^{-1}	< 5	< 5	< 5
Total Alkalinity as $CaCO_3$	mg L^{-1}	31	110	120
Sulphate	mg L^{-1}	9	2,400	2,600
Chloride	mg L^{-1}	20	15,000	18,000
Hardness	mg$CaCO_3$ L^{-1}	120	4,800	6,000
Ionic Balance	%	38	-7.3	-1.9

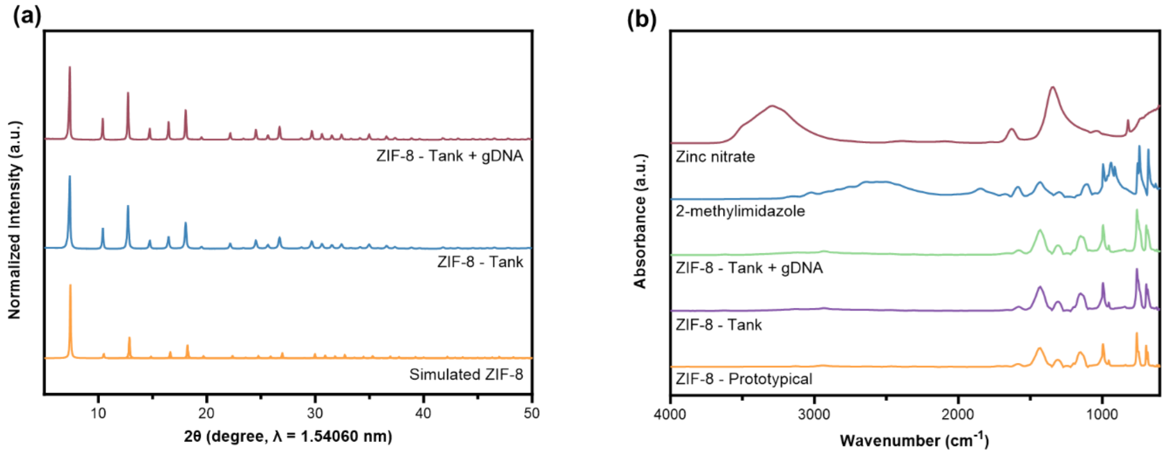

Figure S8. Synthesis of ZIF-8 in tank water with and without addition of salmon gDNA at 90.9 μg mL^{-1}. **(a)** Simulated XRD pattern of pure ZIF-8 compared to synthesised material. **(b)** FTIR spectra comparing the synthesized material to the precursors and pure ZIF-8 without DNA.

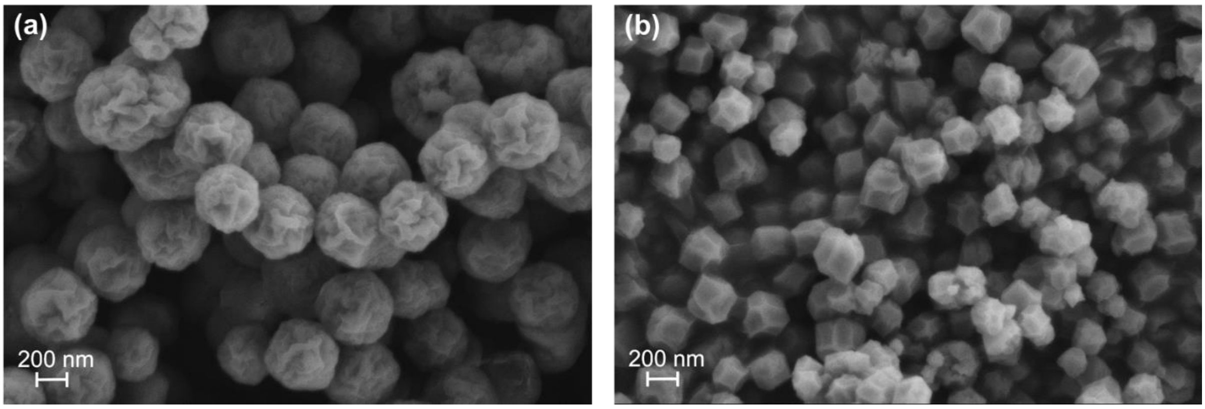

Figure S9. SEM images of ZIF-8 synthesised in (**a**) tank water, (**b**) tank water with 90.9 µg mL^{-1} salmon gDNA at 30,000X magnification.

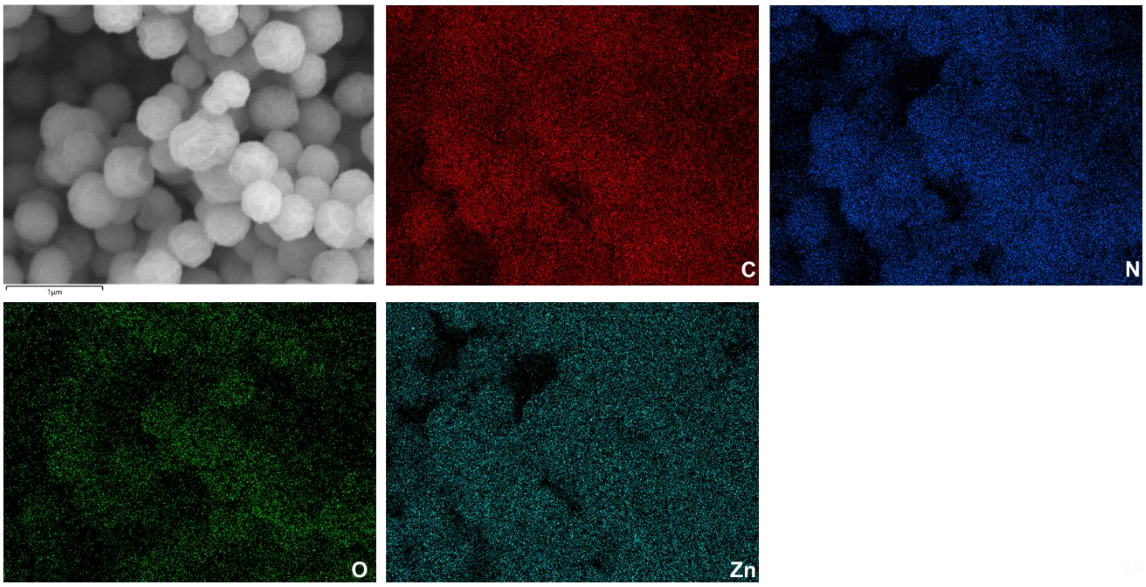

Figure S10. SEM image and EDS elemental mapping images of ZIF-8 synthesized in tank water without added DNA.

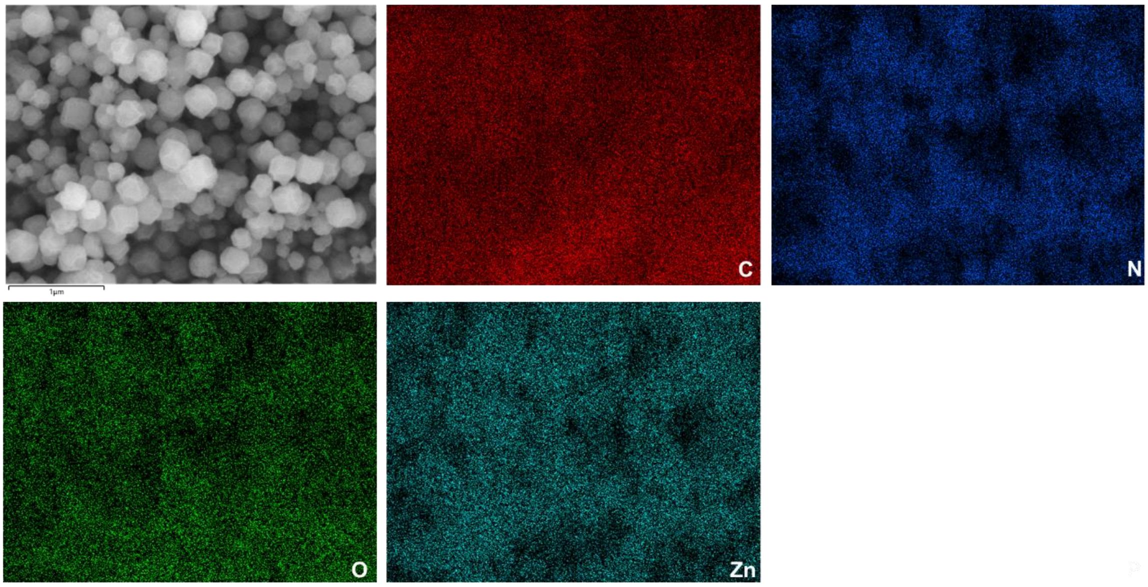

Figure S11. SEM image and EDS elemental mapping images of ZIF-8 synthesized in tank water with salmon DNA at 90.9 μg mL^{-1}

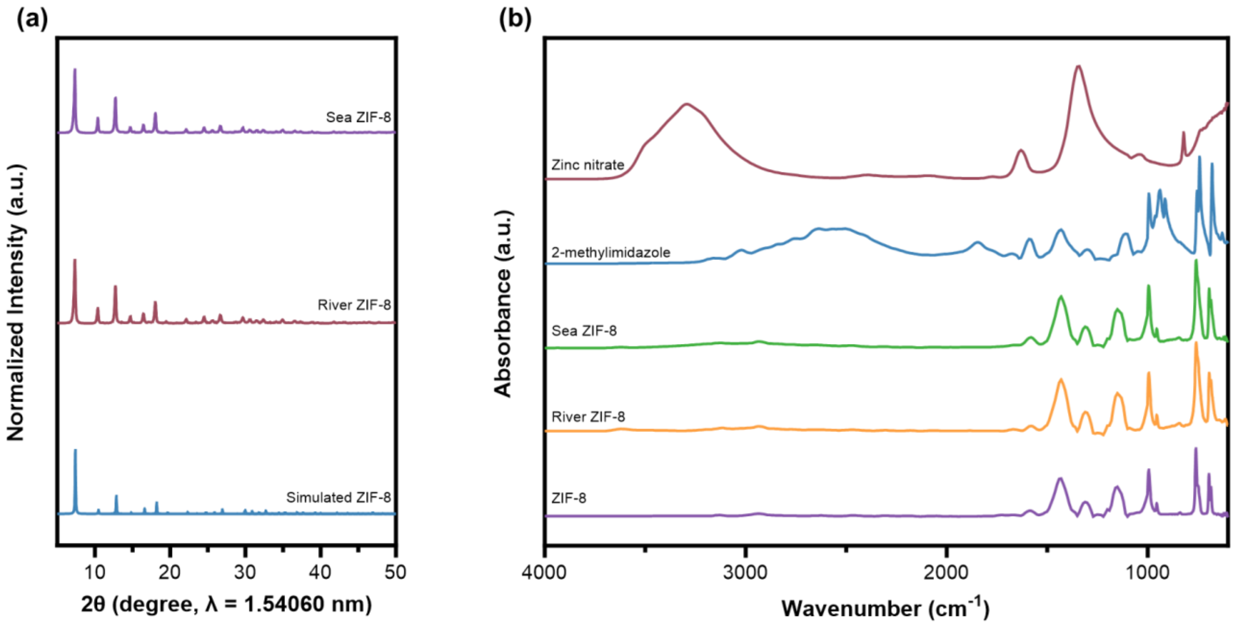

Figure S12. Synthesis of ZIF-8 in river and seawater without added DNA. **(a)** Simulated XRD pattern of pure ZIF-8 compared to synthesised material. **(b)** FTIR spectra comparing the synthesized material to the precursors and pure ZIF-8 without DNA.

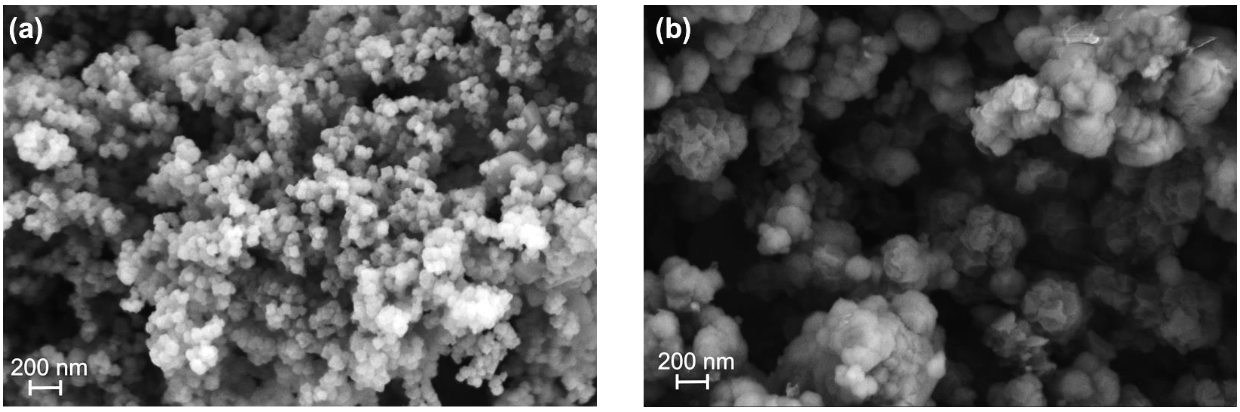

Figure S13. SEM images at 30,000X of ZIF-8 synthesized in (**a**) river or (**b**) sea without additional DNA.

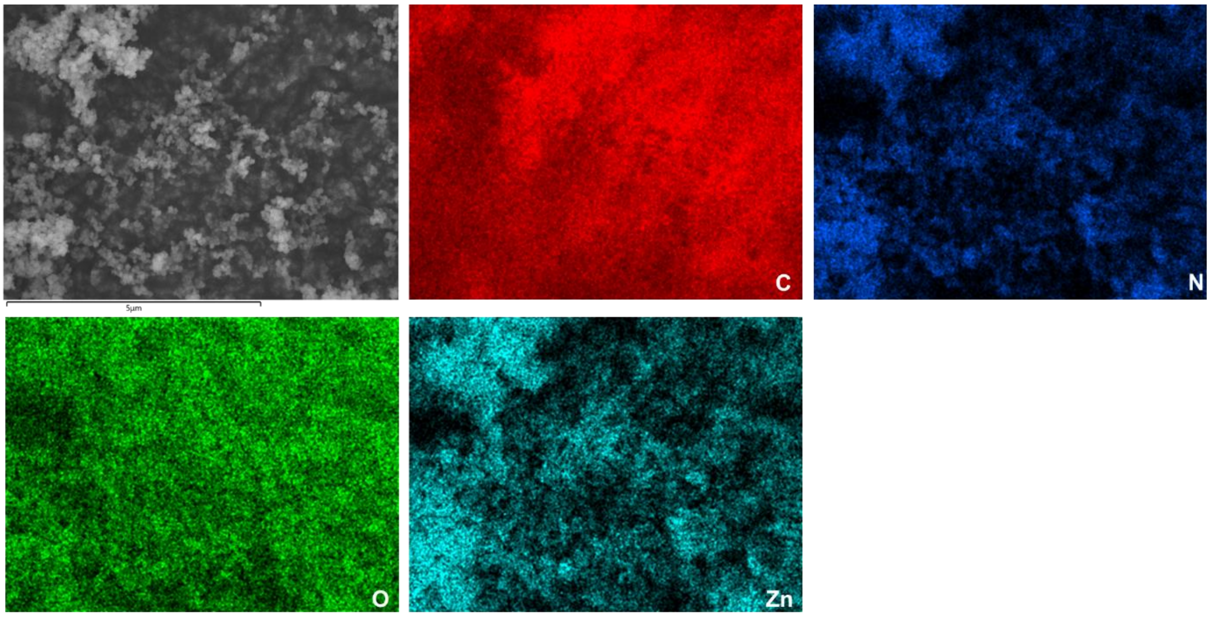

Figure S14. SEM image and EDS elemental mapping images of ZIF-8 synthesized in river water without added gDNA.

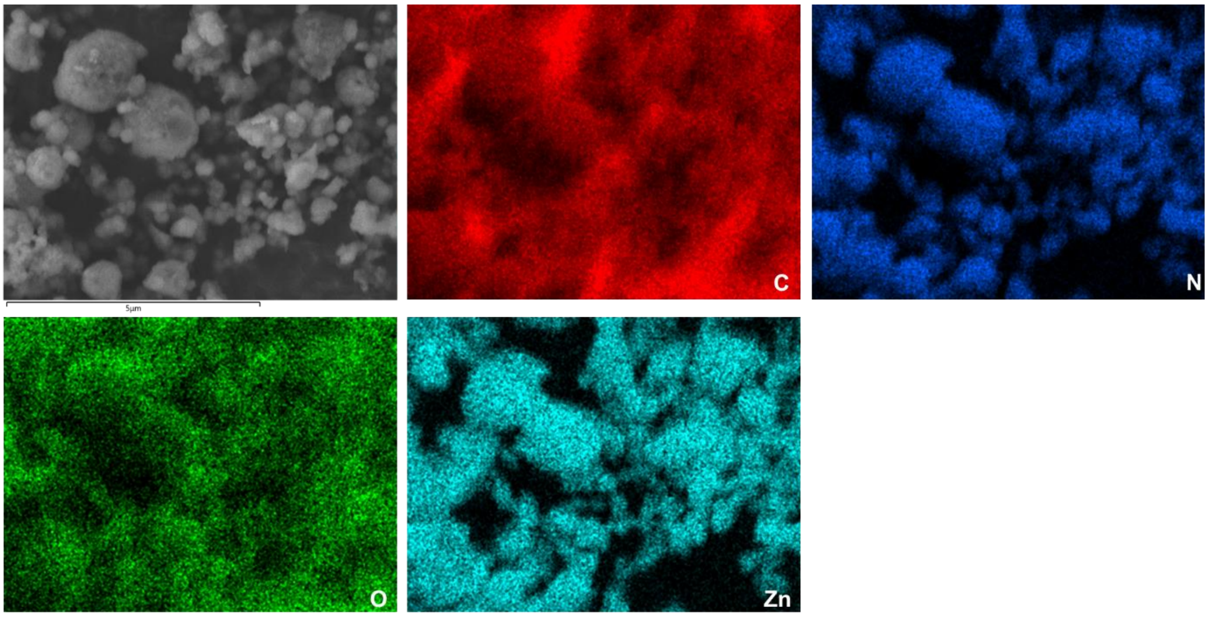

Figure S15. SEM image and EDS elemental mapping images of ZIF-8 synthesized in seawater without added gDNA.

Table S2. Concentration of spiked DNA in Milli-Q water at day 0 after extraction in 100 µL measured via NanoDrop.

Sample	DNA Concentration After Extraction [ng µL^{-1}]	A260/A280 Ratio	A260/A230 Ratio
Free 1	138.8	1.93	2.32
Free 2	143.3	1.90	2.25
Free 3	96.3	1.88	2.13
Captured 1	148.3	1.91	2.28
Captured 2	121.6	1.98	2.31
Captured 3	147.8	1.92	2.24

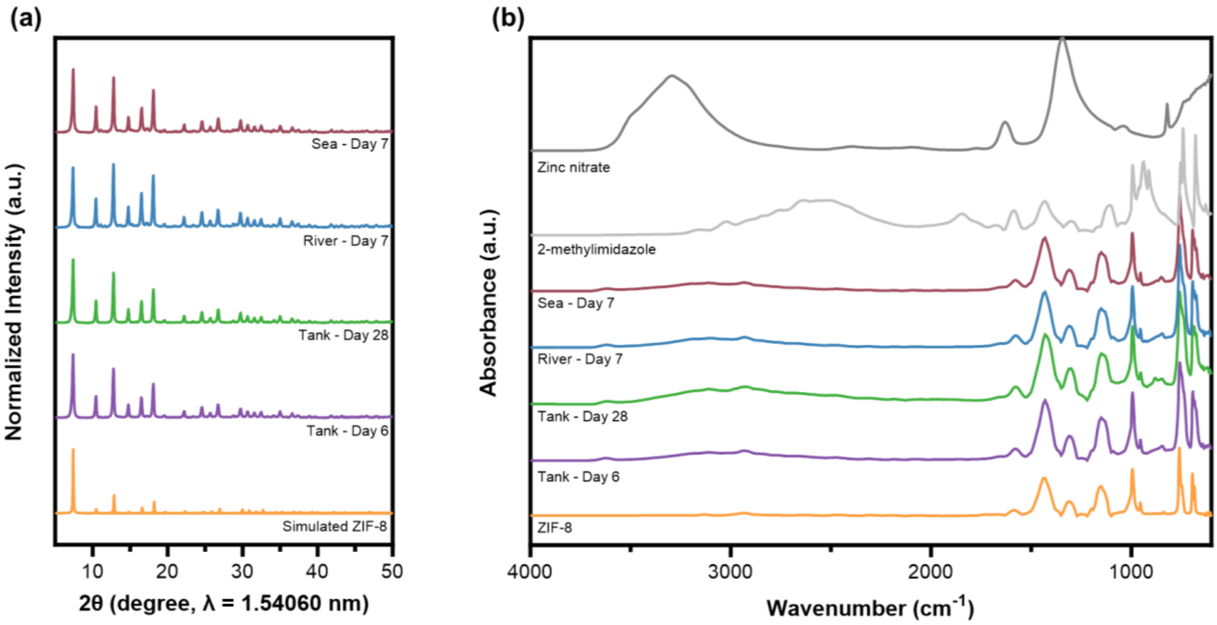

Figure S16. Endpoint characterization of ZIF-8 in tank samples at 6- and 28-days and river or sea samples at 7-days. **a)** Simulated XRD pattern of pure ZIF-8 compared to synthesised material. **(b)** FTIR spectra comparing the synthesized material to the precursors and pure ZIF-8 without DNA.

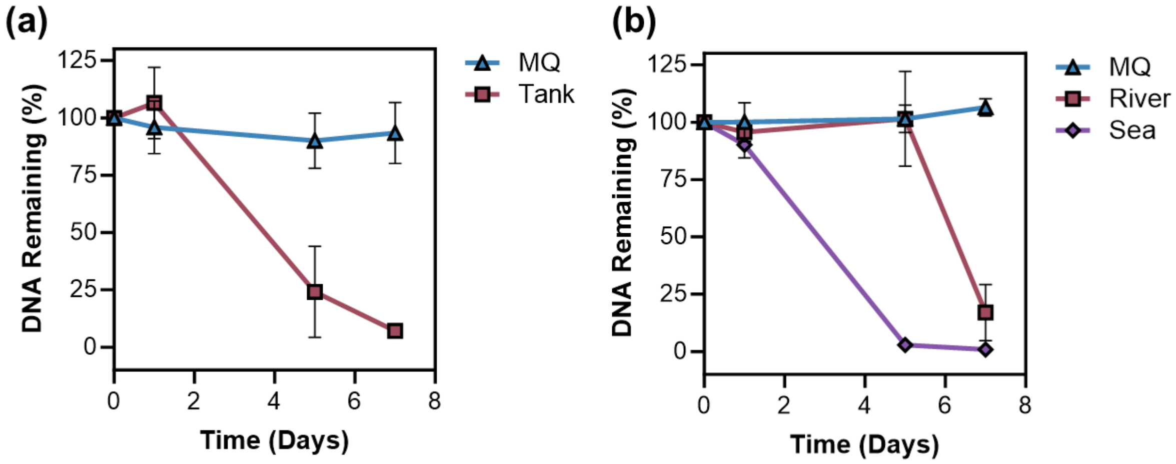

Figure S17. Degradation of free salmon gDNA across all water types. Salmon gDNA was spiked into Milli-Q, (**a**) tank and (**b**) river or seawater at ~67 µg mL^{-1} and the absorbance at 260 nm monitored over a week. Error bars represent the standard deviation for 3 independent experiments in triplicate (n = 9).

Table S3. Concentration of spiked DNA in tank water at day 6 after extraction in 100 µL measured via NanoDrop.

Sample	DNA Concentration After Extraction [ng µL^{-1}]	A260/A280 Ratio	A260/A230 Ratio
Free 1	3.5	4.57	1.20
Free 2	6.1	1.50	2.03
Free 3	3.1	9.91	0.41
Captured 1	222.0	1.89	2.05
Captured 2	335.4	1.85	2.35
Captured 3	850.0	1.84	2.32

Table S4. Concentration of spiked DNA in tank water at day 28 after extraction in 100 µL measured via NanoDrop.

Sample	DNA Concentration After Extraction [ng µL^{-1}]	A260/A280 Ratio	A260/A230 Ratio
Free 1	9.9	1.66	0.86
Free 2	5.12	4.26	1.22
Free 3	10.1	1.53	1.48
Captured 1	772.5	1.86	2.37
Captured 2	516.1	1.84	2.33
Captured 3	361.5	1.85	2.29

Table S5. Concentration of spiked DNA in river water at day 7 after extraction in 100 µL measured via NanoDrop.

Sample	DNA Concentration after Extraction [ng µL^{-1}]	A260/A280 Ratio	A260/A230 Ratio
Free 1	2.8	1.49	0.46
Free 2	2.2	1.50	0.27
Free 3	5.0	1.19	-0.88
Captured 1	696.2	1.87	2.36
Captured 2	573.1	1.83	2.29
Captured 3	343.1	1.83	2.32

Table S6. Concentration of spiked DNA in seawater at day 7 after extraction in 100 µL measured via NanoDrop.

Sample	DNA Concentration after Extraction [ng µL^{-1}]	A260/A280 Ratio	A260/A230 Ratio
Free 1	1.0	0.81	0.14
Free 2	3.5	1.50	0.14
Free 3	6.4	3.01	0.35
Captured 1	216.3	1.83	1.95
Captured 2	490.6	1.83	2.34
Captured 3	495.8	1.82	2.30

Table S7. Results of qPCR assay for goldfish eDNA detection for the 28-day tank samples

Sample	DNA Concentration [ng μL^{-1}]
Free 1	Not detected
Free 2	Not detected
Free 3	Not detected
Captured 1	1.84×10^{-4}
Captured 2	3.72×10^{-5}
Captured 3	9.11×10^{-5}

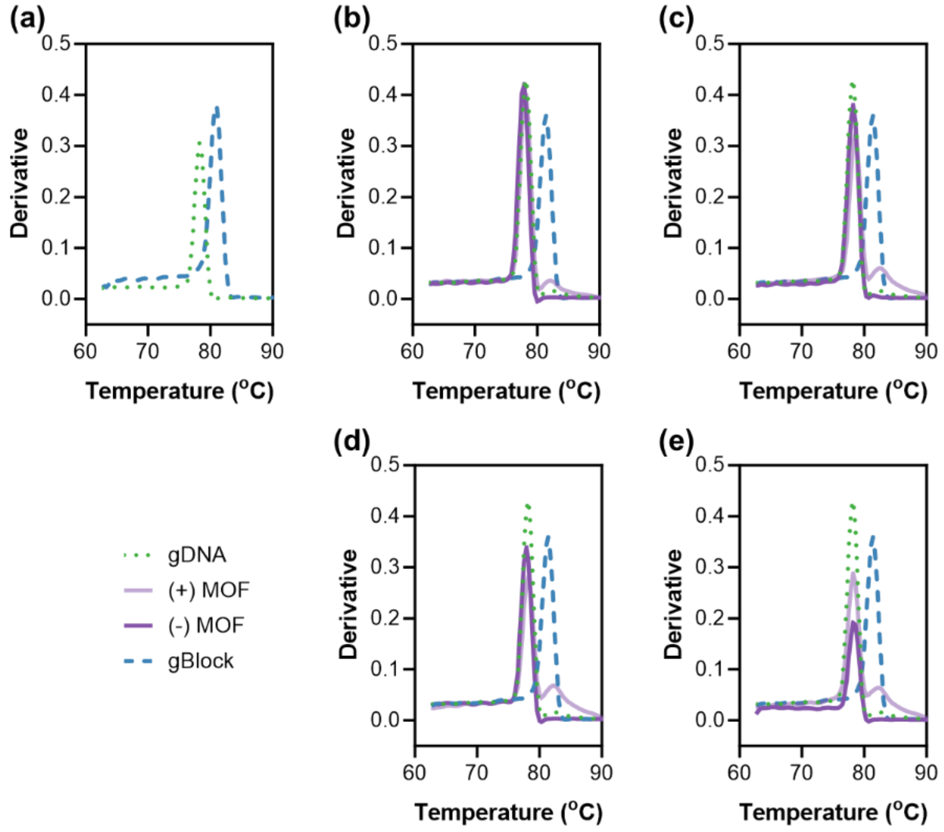

Figure S18. Representative melt curves with comparisons of (**a**) the modified salmon sequence (gBlock) versus the salmon gDNA, (**b**) 6-day tank, (**c**) 28-day tank, 7-day (**d**) river and (**e**) sea samples.

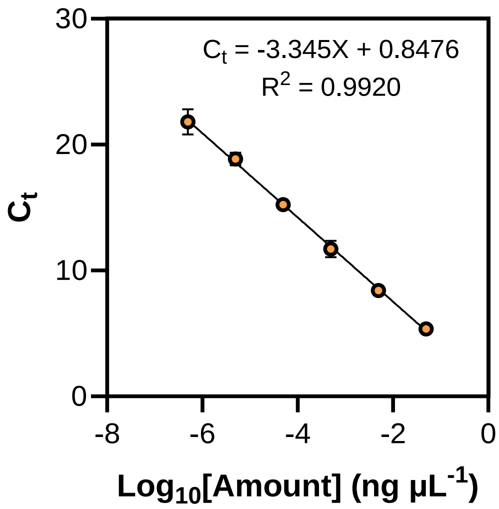

Figure S19. Representative 6-point standard curve using 10-fold serial dilutions of the COI gBlock. The mean of quadruplicate wells is plotted with the error bars representing the standard deviation.
